# Supplementary material for: Altered microRNA Transcriptome in Cultured Human Liver Cells upon Infection with Ebola Virus
Source: Int J Mol Sci. 2021 Apr 6;22(7):3792. doi: 10.3390/ijms22073792 (PMC8038836; doi:10.3390/ijms22073792)
Supplement: Supplementary file 1 [file ijms-22-03792-s001.zip › Supplementary_File/C_ GO_Analysis_Results/16-30nt_go_Reston-96h-Huh7_vs_Control-96h-Huh7_down.mature_mirna_targets/BP_result(Human).html]

| GO.ID | Term | Ontology | Count | Pop.Hits | List.Total | Pop.Total | Fold.Enrichment | Pvalue | FDR | Enrichment.Score | Gene.Ratio | GENES |
| --- | --- | --- | --- | --- | --- | --- | --- | --- | --- | --- | --- | --- |
| GO:0007399 | nervous system development | Biological process | 74 | 2296 | 368 | 17653 | 1.54607493182851 | 7.57642971167205e-05 | 0.314882116573742 | 4.12053540146577 | 0.201086956521739 | FBXO45//MARK1//FZD3//OTP//HIF1A//LRRC38//RTN4//PARD6B//EFNA3//NCAM1//NRAS//CNTN2//VLDLR//UNC5A//TNFRSF8//PCDHB3//BSN//NPTX1//PAX8//CTNS//FOXG1//GRIN2B//CA10//ITGA8//SLC5A3//SFRP1//ZNF804A//LRRK2//BAG5//ACTL6A//TMEM108//PAX5//UCHL5//DIXDC1//DLX5//ALDH1A2//COPS2//HIPK1//FZD2//GNAO1//SMAD1//JAM3//NREP//UBE4B//PRDM12//TG//PLLP//GAL3ST1//KCNIP2//HEYL//VWC2//TLX2//SKIL//PPP3CB//RHOH//CHRNA3//SEMA4G//PPP3CA//LRRTM1//PALM//ABCC8//CTNND2//CPEB3//ACTR2//ZSWIM6//CHRM1//FGF11//KIF2A//NDP//RBFOX1//SCN3B//PURA//SIM1//SMPD1// |
| GO:0030030 | cell projection organization | Biological process | 52 | 1477 | 368 | 17653 | 1.68885961555444 | 0.000129183712532682 | 0.314882116573742 | 3.88879223868674 | 0.141304347826087 | LRRC38//RTN4//PARD6B//EFNA3//NCAM1//NRAS//CNTN2//VLDLR//UNC5A//TNFRSF8//ACTR3//ACTR2//UBXN10//MCIDAS//PARVA//SEPT7//BRK1//SFRP1//ZNF804A//LRRK2//BAG5//FBXO45//RHOH//ARFIP2//ARHGEF6//JAM3//NREP//UBE4B//GNAO1//PRDM12//TMEM108//EMP2//CCDC103//FZD3//PPP3CB//CHRNA3//SEMA4G//SKIL//TLX2//PPP3CA//PALM//NPTX1//CTNND2//CPEB3//DLX5//MAPRE1//PRKAR2B//SH3YL1//EPS8L2//ZSWIM6//GRIN2B//ITGA8// |
| GO:0120036 | plasma membrane bounded cell projection organization | Biological process | 51 | 1444 | 368 | 17653 | 1.69423514091292 | 0.000139678596735358 | 0.314882116573742 | 3.85487013670517 | 0.138586956521739 | LRRC38//RTN4//PARD6B//EFNA3//NCAM1//NRAS//CNTN2//VLDLR//UNC5A//TNFRSF8//ACTR3//ACTR2//UBXN10//MCIDAS//PARVA//SEPT7//BRK1//SFRP1//ZNF804A//LRRK2//BAG5//FBXO45//ARFIP2//ARHGEF6//JAM3//NREP//UBE4B//GNAO1//PRDM12//TMEM108//EMP2//CCDC103//FZD3//PPP3CB//RHOH//CHRNA3//SEMA4G//SKIL//TLX2//PPP3CA//PALM//NPTX1//CTNND2//CPEB3//DLX5//MAPRE1//PRKAR2B//SH3YL1//EPS8L2//ZSWIM6//GRIN2B// |
| GO:0031060 | regulation of histone methylation | Biological process | 7 | 59 | 368 | 17653 | 5.69136882829772 | 0.000217022784339583 | 0.359108280329292 | 3.66349466894634 | 0.0190217391304348 | PAXIP1//PAXBP1//PAX5//PRDM12//OGT//KMT2E//ZFP64// |
| GO:0000904 | cell morphogenesis involved in differentiation | Biological process | 29 | 701 | 368 | 17653 | 1.9844980772809 | 0.000371024887008556 | 0.359108280329292 | 3.43059695850358 | 0.078804347826087 | LRRC38//RTN4//PARD6B//EFNA3//NCAM1//NRAS//CNTN2//VLDLR//UNC5A//TNFRSF8//FBXO45//FERMT2//PARVA//FERMT3//ITGA8//VAMP3//FZD3//PPP3CB//CHRNA3//SEMA4G//SKIL//TLX2//PPP3CA//NPTX1//CTNND2//LRRK2//ACTR2//DLX5//ZSWIM6// |
| GO:0010638 | positive regulation of organelle organization | Biological process | 27 | 637 | 368 | 17653 | 2.0332699133165 | 0.000406174741301357 | 0.359108280329292 | 3.39128708746622 | 0.0733695652173913 | PAXIP1//RHOH//PAXBP1//CDKN1B//MAPRE1//NEK7//TNKS2//ACTR3//ACTR2//ARFIP2//FOXP3//SFRP1//PRDM12//BMP10//OGT//PMAIP1//YWHAB//YWHAZ//CALCOCO2//LRRK2//SEPT7//HIF1A//PRKAA1//UBE2D3//KMT2E//BRK1//PIP4K2C// |
| GO:0007166 | cell surface receptor signaling pathway | Biological process | 88 | 3016 | 368 | 17653 | 1.39965834390497 | 0.000463505032318501 | 0.359108280329292 | 3.33394554631713 | 0.239130434782609 | NRAS//PSMD7//SKP1//FERMT2//CTNND2//TRABD2B//MARK1//NDP//PRKAA1//CXXC4//TNKS2//FZD2//GNAO1//PPP3CA//PPP3CB//FZD3//PTPRT//CD8A//CHRNA3//FAM83B//CAMLG//GDF9//SMAD1//SKIL//GRIN2B//CDKN1B//HEYL//HIPK1//EVC//ITGA5//TLN1//FERMT3//ITGA8//ATP6V1G2//POLR2E//PTBP1//CD46//BMP10//TTK//NREP//HIF1A//LRRTM1//IL4R//IFNK//YWHAZ//FOXP3//SKAP1//SFRP1//DLX5//UBE2D3//HTRA4//RASL11B//VWC2//NOS3//TNFSF4//TNFSF14//TNFRSF8//GPR34//P2RY14//P2RX2//UNC5A//VLDLR//ACTR3//ACTR2//BRK1//PTPN18//TSPAN14//MGRN1//OGT//EFNA3//PAX5//TMEM108//LRRK2//DIXDC1//CNTN2//NCAM1//CXCL11//EBI3//SEMA4G//CLDN18//PMAIP1//RTN4//EMP2//PDIA3//CLEC1A//MAPK4//TSPAN18//CD81// |
| GO:0031056 | regulation of histone modification | Biological process | 10 | 136 | 368 | 17653 | 3.52721387468031 | 0.000569497306653241 | 0.359108280329292 | 3.24450832550657 | 0.0271739130434783 | PAXIP1//ZFP64//PAXBP1//FOXP3//PAX5//PRDM12//OGT//LRRK2//KMT2E//PARG// |
| GO:0010498 | proteasomal protein catabolic process | Biological process | 21 | 453 | 368 | 17653 | 2.22377987330838 | 0.000573016419297005 | 0.359108280329292 | 3.24183293351617 | 0.0570652173913043 | UCHL5//NUDT15//UBE4B//PSMD7//UBE2E1//BUB3//FBXL3//SKP1//OGT//BAG5//LRRK2//RNF217//DNAJB12//MAEA//UBXN2B//UBXN2A//FBXO45//RLIM//UBE2D3//ALAD//PMAIP1// |
| GO:0030182 | neuron differentiation | Biological process | 45 | 1303 | 368 | 17653 | 1.65668065334179 | 0.000574062591806899 | 0.359108280329292 | 3.24104075255959 | 0.122282608695652 | LRRC38//RTN4//PARD6B//EFNA3//NCAM1//NRAS//CNTN2//VLDLR//UNC5A//TNFRSF8//SFRP1//ZNF804A//LRRK2//BAG5//OTP//DLX5//FBXO45//JAM3//NREP//UBE4B//GNAO1//PRDM12//TMEM108//FZD3//KCNIP2//DIXDC1//HEYL//VWC2//SKIL//PPP3CB//RHOH//CHRNA3//SEMA4G//TLX2//PPP3CA//FZD2//NPTX1//CTNND2//CPEB3//ACTR2//HIF1A//ZSWIM6//HIPK1//ALDH1A2//COPS2// |
| GO:0031175 | neuron projection development | Biological process | 35 | 936 | 368 | 17653 | 1.79375406447417 | 0.000611560729851099 | 0.359108280329292 | 3.21356040971578 | 0.0951086956521739 | LRRC38//RTN4//PARD6B//EFNA3//NCAM1//NRAS//CNTN2//VLDLR//UNC5A//TNFRSF8//SFRP1//ZNF804A//LRRK2//BAG5//FBXO45//JAM3//NREP//FZD3//PPP3CB//RHOH//CHRNA3//SEMA4G//SKIL//TLX2//PPP3CA//NPTX1//CTNND2//CPEB3//ACTR2//DLX5//TMEM108//ZSWIM6//UBE4B//GNAO1//PRDM12// |
| GO:0031062 | positive regulation of histone methylation | Biological process | 5 | 34 | 368 | 17653 | 7.05442774936061 | 0.000648143681493416 | 0.359108280329292 | 3.18832870838441 | 0.0135869565217391 | PAXIP1//PRDM12//OGT//KMT2E//PAXBP1// |
| GO:0048812 | neuron projection morphogenesis | Biological process | 26 | 628 | 368 | 17653 | 1.98602360841872 | 0.000733669470718108 | 0.359108280329292 | 3.13449955230933 | 0.0706521739130435 | LRRC38//RTN4//PARD6B//EFNA3//NCAM1//NRAS//CNTN2//VLDLR//UNC5A//TNFRSF8//FBXO45//FZD3//PPP3CB//CHRNA3//SEMA4G//SKIL//TLX2//PPP3CA//NPTX1//CTNND2//LRRK2//ACTR2//DLX5//TMEM108//ZSWIM6//RHOH// |
| GO:0002823 | negative regulation of adaptive immune response based on somatic recombination of immune receptors built from immunoglobulin superfamily domains | Biological process | 5 | 35 | 368 | 17653 | 6.85287267080745 | 0.000743385468669243 | 0.359108280329292 | 3.12878593253064 | 0.0135869565217391 | PPP3CB//FOXP3//IL4R//TNFSF4//NDFIP1// |
| GO:0048667 | cell morphogenesis involved in neuron differentiation | Biological process | 24 | 567 | 368 | 17653 | 2.03048079135036 | 0.000861053318303351 | 0.369314768595629 | 3.0649699552502 | 0.0652173913043478 | LRRC38//RTN4//PARD6B//EFNA3//NCAM1//NRAS//CNTN2//VLDLR//UNC5A//TNFRSF8//FBXO45//FZD3//PPP3CB//CHRNA3//SEMA4G//SKIL//TLX2//PPP3CA//NPTX1//CTNND2//LRRK2//ACTR2//DLX5//ZSWIM6// |
| GO:0042758 | long-chain fatty acid catabolic process | Biological process | 3 | 10 | 368 | 17653 | 14.3910326086957 | 0.000966983888159384 | 0.369314768595629 | 3.01458076205119 | 0.00815217391304348 | CYP4F3//CYP4F2//ACADL// |
| GO:0120039 | plasma membrane bounded cell projection morphogenesis | Biological process | 26 | 642 | 368 | 17653 | 1.94271468237844 | 0.00100948799480254 | 0.369314768595629 | 2.99589884147717 | 0.0706521739130435 | LRRC38//RTN4//PARD6B//EFNA3//NCAM1//NRAS//CNTN2//VLDLR//UNC5A//TNFRSF8//FBXO45//FZD3//PPP3CB//LRRK2//RHOH//CHRNA3//SEMA4G//SKIL//TLX2//PPP3CA//NPTX1//CTNND2//ACTR2//DLX5//TMEM108//ZSWIM6// |
| GO:0048858 | cell projection morphogenesis | Biological process | 26 | 644 | 368 | 17653 | 1.93668140696732 | 0.00105546016406114 | 0.369314768595629 | 2.97655815350261 | 0.0706521739130435 | LRRC38//RTN4//PARD6B//EFNA3//NCAM1//NRAS//CNTN2//VLDLR//UNC5A//TNFRSF8//FBXO45//FZD3//PPP3CB//LRRK2//RHOH//CHRNA3//SEMA4G//SKIL//TLX2//PPP3CA//NPTX1//CTNND2//ACTR2//DLX5//TMEM108//ZSWIM6// |
| GO:0002820 | negative regulation of adaptive immune response | Biological process | 5 | 38 | 368 | 17653 | 6.31185640732265 | 0.00109216255684054 | 0.369314768595629 | 2.96171271668189 | 0.0135869565217391 | PPP3CB//FOXP3//IL4R//TNFSF4//NDFIP1// |
| GO:0007223 | Wnt signaling pathway, calcium modulating pathway | Biological process | 5 | 38 | 368 | 17653 | 6.31185640732265 | 0.00109216255684054 | 0.369314768595629 | 2.96171271668189 | 0.0135869565217391 | FZD2//GNAO1//PPP3CA//PPP3CB//FZD3// |
| GO:0048699 | generation of neurons | Biological process | 47 | 1437 | 368 | 17653 | 1.56895971377568 | 0.001363677733115 | 0.419832270445007 | 2.86528825084245 | 0.127717391304348 | FBXO45//MARK1//FZD3//OTP//HIF1A//LRRC38//RTN4//PARD6B//EFNA3//NCAM1//NRAS//CNTN2//VLDLR//UNC5A//TNFRSF8//SFRP1//ZNF804A//LRRK2//BAG5//DLX5//HIPK1//FZD2//ALDH1A2//COPS2//JAM3//NREP//UBE4B//GNAO1//PRDM12//TMEM108//KCNIP2//DIXDC1//HEYL//VWC2//SKIL//PPP3CB//RHOH//CHRNA3//SEMA4G//TLX2//PPP3CA//ABCC8//NPTX1//CTNND2//CPEB3//ACTR2//ZSWIM6// |
| GO:0050789 | regulation of biological process | Biological process | 271 | 11709 | 368 | 17653 | 1.11024848035885 | 0.00136571195472278 | 0.419832270445007 | 2.86464088898955 | 0.736413043478261 | CDKN1B//CPEB3//HEYL//KCNIP3//PAX5//FOXP3//RLIM//SKIL//UBE2D3//VLDLR//BHLHE40//COPS2//PAXBP1//LRRK2//GRIN2B//SMAD1//NCAM1//NRAS//MAPK4//PSMD7//YWHAB//PNRC1//PRKAA1//PROK1//CD81//EIF4A2//RBFOX1//PTBP1//PAXIP1//AGTR1//HTRA4//TNFSF4//PMAIP1//CAMLG//PPP3CB//VAMP3//ALDH1A2//HIF1A//EMP2//SFRP1//EPAS1//NOS3//OTP//FZD3//LRRTM1//SKP1//IL4R//CREB3L3//CXCL11//SKAP1//NDFIP1//CHRM1//COPS5//NUP214//FERMT2//CTNND2//TRABD2B//MARK1//NDP//CXXC4//TNKS2//FZD2//GNAO1//PPP3CA//FAM13A//RALBP1//PRDM4//PTPRT//AKAP10//CHRNA3//ARHGAP36//FGF11//PDIA3//KCNIP2//IGSF1//MRC1//RASL11B//SMPD1//ABCC8//TG//YWHAZ//OGT//ACTL6A//TNFSF14//TNFRSF8//SH3BP5//CD8A//CARHSP1//PRKAR2B//POLR2M//OXSR1//PPP1R3F//TXNDC5//SH3BGRL3//MCIDAS//PARG//KRBOX1//ZBTB6//ZNF618//HIPK1//EWSR1//ZBTB43//ATP1B4//MYCBP//NRBF2//HMGB3//RHOH//MLF1//UCHL5//PFDN1//ASF1B//ZFP64//IFNK//ZNF124//ZNF322//ELK1//FOXG1//HOXC9//PURA//CREBZF//PRDM12//SIM1//VEZF1//CREB5//RBM3//EIF4G2//CEP85//CD46//KMT2E//TTK//BUB3//CLEC1A//TSPAN14//TSPAN18//FAM83B//GDF9//GPR160//GNAZ//GPR34//RIC8B//ARHGEF6//P2RY14//GPR3//PALM//EVC//ITGA5//TLN1//FERMT3//ITGA8//DDAH1//ARFIP2//RHOF//RALGPS2//RRAS//EPS8L2//MAEA//NEK7//TTC28//SLC25A5//BRK1//PGGT1B//C18ORF54//LDOC1//DEC1//ATP6V1G2//PARVA//NUDT15//POLR2E//UBE4B//CASP7//PYURF//PPP1R2//MAPRE1//SCN3B//UBXN2A//EBI3//BMP10//DRAM1//PIP4K2C//TP53INP2//DLAT//P2RX2//ZNF804A//CAPN7//RTN4//TRDN//PAX8//CTNS//MICAL2//VWC2//STARD4//MEST//ABHD5//INSIG2//RRP1B//ZCCHC9//PPP1R26//A2ML1//CNTN2//BAG5//LARP1//MGRN1//TNFAIP8L3//SEPT7//EFNA3//DLX5//NISCH//NREP//MEX3B//CPLX1//SYT14//STON2//KIF2A//BEX4//PARD6B//SEMA4G//STATH//CTH//TBC1D22B//CYP4F2//RNF217//RNF135//RABL3//DIXDC1//RFK//WDFY1//ACTR3//ACTR2//SCN1A//SCN7A//KCNQ4//KDELR3//GOSR2//UNC5A//PTPN18//FBXL3//SHQ1//RNF149//WDR91//SLC5A3//UBXN2B//PPIH//IDI1//ACADL//CLDN18//TLX2//TMPRSS2//CGNL1//NOVA1//TMEM108//BEST1//JPH1//JAM3//BSN//LYPD6//SH3YL1//BAG2//LSM11//SNRPA//PEX5//CALCOCO2//ALAD//ME2//AOC3//ZSWIM6//NUCB1//LRRC38//SESTD1//FAM168A//GRIPAP1//RNF169// |
| GO:0048666 | neuron development | Biological process | 37 | 1060 | 368 | 17653 | 1.67442832239541 | 0.0014912803551621 | 0.438501262693969 | 2.82644070312363 | 0.10054347826087 | LRRC38//RTN4//PARD6B//EFNA3//NCAM1//NRAS//CNTN2//VLDLR//UNC5A//TNFRSF8//SFRP1//ZNF804A//LRRK2//BAG5//FBXO45//JAM3//NREP//UBE4B//GNAO1//PRDM12//TMEM108//FZD3//KCNIP2//PPP3CB//RHOH//CHRNA3//SEMA4G//SKIL//TLX2//PPP3CA//FZD2//NPTX1//CTNND2//CPEB3//ACTR2//DLX5//ZSWIM6// |
| GO:0032990 | cell part morphogenesis | Biological process | 26 | 663 | 368 | 17653 | 1.88118073316283 | 0.00159096918995451 | 0.448321859652598 | 2.79833823063771 | 0.0706521739130435 | LRRC38//RTN4//PARD6B//EFNA3//NCAM1//NRAS//CNTN2//VLDLR//UNC5A//TNFRSF8//FBXO45//FZD3//PPP3CB//LRRK2//RHOH//CHRNA3//SEMA4G//SKIL//TLX2//PPP3CA//NPTX1//CTNND2//ACTR2//DLX5//TMEM108//ZSWIM6// |
| GO:0048302 | regulation of isotype switching to IgG isotypes | Biological process | 3 | 12 | 368 | 17653 | 11.992527173913 | 0.00171865150998136 | 0.448734879707605 | 2.76481217603858 | 0.00815217391304348 | PAXIP1//FOXP3//NDFIP1// |
| GO:0031058 | positive regulation of histone modification | Biological process | 7 | 83 | 368 | 17653 | 4.04567181770561 | 0.00172513778979709 | 0.448734879707605 | 2.76317621133931 | 0.0190217391304348 | PAXIP1//PAXBP1//FOXP3//PRDM12//OGT//LRRK2//KMT2E// |
| GO:0001776 | leukocyte homeostasis | Biological process | 7 | 84 | 368 | 17653 | 3.99750905797101 | 0.00184899422863119 | 0.461815791496735 | 2.7330644444263 | 0.0190217391304348 | JAM3//FOXP3//HIF1A//SKIL//PMAIP1//PPP3CB//TNFSF14// |
| GO:0002260 | lymphocyte homeostasis | Biological process | 6 | 63 | 368 | 17653 | 4.5685817805383 | 0.00196837865247635 | 0.461815791496735 | 2.70589135363694 | 0.016304347826087 | FOXP3//HIF1A//PMAIP1//PPP3CB//TNFSF14//SKIL// |
| GO:0051128 | regulation of cellular component organization | Biological process | 70 | 2403 | 368 | 17653 | 1.3973814434855 | 0.00210677318204205 | 0.461815791496735 | 2.67638221858743 | 0.190217391304348 | PAXIP1//AGTR1//HTRA4//LRRTM1//TTK//BUB3//CDKN1B//BRK1//FERMT2//PALM//RHOF//PARVA//SFRP1//ZNF804A//LRRK2//TNFRSF8//BAG5//SEPT7//STON2//EIF4G2//GDF9//BMP10//RTN4//EPS8L2//RHOH//PARG//ZFP64//PAXBP1//FOXP3//MAPRE1//CNTN2//TBC1D22B//NEK7//TNKS2//TRABD2B//PMAIP1//DIXDC1//ACTR3//ACTR2//ARFIP2//GPR3//PPP3CA//CEP85//CHRNA3//SEMA4G//SKIL//TLX2//PAX5//PRDM12//CPEB3//OGT//JAM3//VLDLR//SH3YL1//YWHAB//YWHAZ//SLC25A5//PEX5//CALCOCO2//IL4R//TNFSF14//ZSWIM6//GRIN2B//HIF1A//PRKAA1//UBE2D3//ABCC8//GRIPAP1//KMT2E//PIP4K2C// |
| GO:0033036 | macromolecule localization | Biological process | 83 | 2955 | 368 | 17653 | 1.34738376370191 | 0.00218752035361637 | 0.461815791496735 | 2.66004789742695 | 0.22554347826087 | PDIA3//SPRN//PPP3CA//NUP214//SLC27A4//PMAIP1//AGTR1//AKAP10//MAPRE1//PLLP//CD81//U2AF1//YWHAB//YWHAZ//GOLGA7B//KDELR3//GOSR2//TIMM23//TOMM34//ABCA13//PCTP//ANO3//VLDLR//AP1S1//SNX31//ARFIP2//RABL3//KCNIP3//TBC1D22B//MLPH//TLN1//SLC25A5//PPP3CB//ABCC8//OTOR//STARD4//MEST//ABHD5//VPS53//SCAMP1//TNFAIP8L3//PEX5//CPLX1//TOMM40L//CYP4F2//LRRK2//ATP6V1G2//EMP2//TTK//SEPT7//LRRTM1//BSN//HIF1A//SYS1//VAMP3//SPCS2//ITGA8//SFRP1//RBFOX1//FOXP3//TNFSF4//NDFIP1//SKP1//TULP4//TNKS2//CNTN2//FERMT2//PALM//SCN3B//TSPAN14//SKAP1//IL4R//CHRM1//FABP2//GRIPAP1//CLDN18//NUCB1//TP53INP2//PRKAA1//UBE2D3//SHQ1//RTN4//GDF9// |
| GO:0048291 | isotype switching to IgG isotypes | Biological process | 3 | 13 | 368 | 17653 | 11.070025083612 | 0.00219991232291127 | 0.461815791496735 | 2.65759462755716 | 0.00815217391304348 | FOXP3//NDFIP1//PAXIP1// |
| GO:1902692 | regulation of neuroblast proliferation | Biological process | 4 | 27 | 368 | 17653 | 7.10668276972625 | 0.00223095214681623 | 0.461815791496735 | 2.65150974508888 | 0.0108695652173913 | OTP//HIF1A//FZD3//LRRK2// |
| GO:1902275 | regulation of chromatin organization | Biological process | 10 | 163 | 368 | 17653 | 2.94295145372099 | 0.00225342615989831 | 0.461815791496735 | 2.6471566682893 | 0.0271739130434783 | PAXIP1//PARG//ZFP64//PAXBP1//FOXP3//PAX5//PRDM12//OGT//LRRK2//KMT2E// |
| GO:0048731 | system development | Biological process | 124 | 4760 | 368 | 17653 | 1.24964148702959 | 0.00236014348549238 | 0.469460305658381 | 2.62706159318951 | 0.33695652173913 | CLEC3A//DLX5//EVC//OTOR//EPAS1//HIF1A//ITGA5//NOS3//RTN4//VEZF1//JAM3//PROK1//ALDH1A2//PAXIP1//HIPK1//PAX8//ITGA8//SMAD1//SFRP1//SIM1//SEMA4G//FBXO45//MARK1//FZD3//AGTR1//CDKN1B//NDP//LDOC1//PPP3CB//CCDC103//MICAL2//PARVA//OTP//CTNS//STON2//MLF1//FOXP3//RRAS//NDFIP1//FZD2//HEYL//UBE4B//KRT2//ACTL6A//PAX5//CHRM1//CHRNA3//FGF11//KIF2A//RBFOX1//SCN3B//PCDHB3//PURA//SMPD1//VLDLR//LRRC38//PARD6B//EFNA3//NCAM1//NRAS//CNTN2//UNC5A//TNFRSF8//BSN//NPTX1//FOXG1//GRIN2B//CA10//SLC5A3//BMP10//JPH1//PAXBP1//MEA1//ZNF804A//LRRK2//BAG5//ABCC8//TMEM108//UCHL5//DIXDC1//COPS2//IFNK//RHOH//KMT2E//STATH//TG//GNAO1//NREP//PRDM12//KRTAP1-1//INSIG2//KCNQ4//PLLP//GAL3ST1//GPD2//MAEA//TNFSF4//CD46//SLC27A4//CD8A//KCNIP2//HMGB3//IL4R//VWC2//EMP2//DDAH1//TLX2//CLDN18//PRKAA1//SKIL//HOXC9//P2RX2//C11ORF88//PPP3CA//LRRTM1//PSMD7//PALM//CTNND2//CPEB3//ACTR2//BFSP1//ZSWIM6//CTH//GDF9// |
| GO:0048468 | cell development | Biological process | 61 | 2045 | 368 | 17653 | 1.43089321781652 | 0.00246552355286467 | 0.476409593943536 | 2.60809084434749 | 0.165760869565217 | GDF9//HIF1A//SEMA4G//VEZF1//OTP//FZD3//KRT2//STRBP//JAM3//LRRC38//RTN4//PARD6B//EFNA3//NCAM1//NRAS//CNTN2//VLDLR//UNC5A//TNFRSF8//SFRP1//ZNF804A//LRRK2//BAG5//HEYL//ALDH1A2//FBXO45//NREP//UBE4B//GNAO1//PRDM12//TMEM108//FERMT2//PARVA//FERMT3//ITGA8//VAMP3//MAEA//KCNIP2//BMP10//DIXDC1//VWC2//EPAS1//BFSP1//MCMDC2//SKIL//PPP3CB//P2RX2//C11ORF88//PPP3CA//RHOH//CHRNA3//TLX2//FZD2//ABCC8//NPTX1//CTNND2//CPEB3//ACTR2//DLX5//ZSWIM6//CLDN18// |
| GO:0045620 | negative regulation of lymphocyte differentiation | Biological process | 5 | 46 | 368 | 17653 | 5.21414224952741 | 0.00260372036547314 | 0.487718297760816 | 2.584405659986 | 0.0135869565217391 | HMGB3//SFRP1//TNFSF4//IL4R//FOXP3// |
| GO:1905269 | positive regulation of chromatin organization | Biological process | 7 | 90 | 368 | 17653 | 3.73100845410628 | 0.00274341851766461 | 0.487718297760816 | 2.56170793426657 | 0.0190217391304348 | PAXIP1//PAXBP1//FOXP3//PRDM12//OGT//LRRK2//KMT2E// |
| GO:0045623 | negative regulation of T-helper cell differentiation | Biological process | 3 | 14 | 368 | 17653 | 10.2793090062112 | 0.00275690648423746 | 0.487718297760816 | 2.55957796514865 | 0.00815217391304348 | IL4R//TNFSF4//FOXP3// |
| GO:1905114 | cell surface receptor signaling pathway involved in cell-cell signaling | Biological process | 24 | 620 | 368 | 17653 | 1.85690743338008 | 0.00281251125427648 | 0.487718297760816 | 2.55090573104577 | 0.0652173913043478 | FERMT2//CTNND2//TRABD2B//MARK1//NDP//PRKAA1//SKP1//CXXC4//TNKS2//FZD2//GNAO1//PPP3CA//PPP3CB//FZD3//FOXP3//SFRP1//LRRK2//DIXDC1//PSMD7//CHRNA3//P2RX2//GRIN2B//DLX5//TMEM108// |
| GO:0051130 | positive regulation of cellular component organization | Biological process | 40 | 1221 | 368 | 17653 | 1.5715023323719 | 0.00303779214992979 | 0.512228690019387 | 2.51744194450438 | 0.108695652173913 | PAXIP1//BRK1//ZNF804A//RHOH//PAXBP1//CDKN1B//MAPRE1//NEK7//TNKS2//TRABD2B//PMAIP1//ACTR3//ACTR2//ARFIP2//FOXP3//PPP3CA//RTN4//SKIL//PALM//SFRP1//PRDM12//LRRTM1//BMP10//CPEB3//OGT//VLDLR//EPS8L2//LRRK2//YWHAB//YWHAZ//CALCOCO2//IL4R//TNFSF14//SEPT7//HIF1A//PRKAA1//UBE2D3//ABCC8//KMT2E//PIP4K2C// |
| GO:0051341 | regulation of oxidoreductase activity | Biological process | 7 | 92 | 368 | 17653 | 3.64989957466919 | 0.00310533436208707 | 0.512228690019387 | 2.50789163098132 | 0.0190217391304348 | AGTR1//RFK//DDAH1//NOS3//HIF1A//AOC3//LRRK2// |
| GO:0045191 | regulation of isotype switching | Biological process | 4 | 30 | 368 | 17653 | 6.39601449275362 | 0.00331684370275969 | 0.52156352627319 | 2.47927499294488 | 0.0108695652173913 | PAXIP1//FOXP3//NDFIP1//TNFSF4// |
| GO:0000902 | cell morphogenesis | Biological process | 34 | 997 | 368 | 17653 | 1.63589136976146 | 0.00333914629281071 | 0.52156352627319 | 2.47636455346176 | 0.0923913043478261 | LRRC38//RTN4//PARD6B//EFNA3//NCAM1//NRAS//CNTN2//VLDLR//UNC5A//TNFRSF8//FERMT2//PALM//RHOF//PARVA//SEPT7//FBXO45//FERMT3//ITGA8//VAMP3//FZD3//PPP3CB//LRRK2//RHOH//CHRNA3//SEMA4G//SKIL//TLX2//PPP3CA//NPTX1//CTNND2//ACTR2//DLX5//TMEM108//ZSWIM6// |
| GO:0043371 | negative regulation of CD4-positive, alpha-beta T cell differentiation | Biological process | 3 | 15 | 368 | 17653 | 9.59402173913043 | 0.00339328628656223 | 0.52156352627319 | 2.46937949802385 | 0.00815217391304348 | IL4R//TNFSF4//FOXP3// |
| GO:0016482 | cytosolic transport | Biological process | 9 | 146 | 368 | 17653 | 2.95706149493746 | 0.00356766156790677 | 0.5361798929723 | 2.44761634996903 | 0.0244565217391304 | SYS1//AP1S1//VPS53//VAMP3//GOSR2//EMP2//WDR91//LRRK2//ACTR2// |
| GO:0070647 | protein modification by small protein conjugation or removal | Biological process | 37 | 1119 | 368 | 17653 | 1.58614300423515 | 0.00372283429430721 | 0.539099533013462 | 2.42912629407158 | 0.10054347826087 | UBE4B//RNF217//MGRN1//FBXL3//RLIM//PSMD7//SKP1//UBE2D3//UBE2E1//TNKS2//COPS5//COPS2//MAEA//FBXO45//EPAS1//RNF169//RNF149//HIF1A//KLHL7//TULP4//KLHL14//PEX5//TRIM7//RNF135//KBTBD6//USP54//SMAD1//UCHL5//USP53//OGT//ACTL6A//NUP214//UBXN2A//BAG5//LRRK2//PAXIP1//NDFIP1// |
| GO:0050869 | negative regulation of B cell activation | Biological process | 4 | 31 | 368 | 17653 | 6.18969144460028 | 0.00374651457217695 | 0.539099533013462 | 2.42637257386163 | 0.0108695652173913 | HMGB3//SFRP1//FOXP3//NDFIP1// |
| GO:0061564 | axon development | Biological process | 20 | 497 | 368 | 17653 | 1.93038666783309 | 0.00402704066979727 | 0.561792152611332 | 2.3950139843535 | 0.0543478260869565 | LRRC38//RTN4//PARD6B//EFNA3//NCAM1//NRAS//CNTN2//VLDLR//UNC5A//TNFRSF8//FBXO45//JAM3//NREP//FZD3//PPP3CB//SEMA4G//SKIL//NPTX1//DLX5//ZSWIM6// |
| GO:0035587 | purinergic receptor signaling pathway | Biological process | 4 | 32 | 368 | 17653 | 5.99626358695652 | 0.00421239540254422 | 0.561792152611332 | 2.3754708698979 | 0.0108695652173913 | GPR34//P2RY14//P2RX2//CNTN2// |
| GO:0042472 | inner ear morphogenesis | Biological process | 7 | 98 | 368 | 17653 | 3.42643633540373 | 0.00441221942869435 | 0.561792152611332 | 2.35534289734213 | 0.0190217391304348 | FZD2//DLX5//INSIG2//PAX8//FZD3//ITGA8//KCNQ4// |
| GO:0016567 | protein ubiquitination | Biological process | 29 | 827 | 368 | 17653 | 1.68214407759844 | 0.00447599205169575 | 0.561792152611332 | 2.34911069334738 | 0.078804347826087 | UBE4B//RNF217//MGRN1//FBXL3//RLIM//PSMD7//SKP1//UBE2D3//UBE2E1//TNKS2//UBXN2A//OGT//BAG5//LRRK2//PAXIP1//NDFIP1//MAEA//FBXO45//EPAS1//RNF169//RNF149//HIF1A//KLHL7//TULP4//KLHL14//PEX5//TRIM7//RNF135//KBTBD6// |
| GO:0007611 | learning or memory | Biological process | 12 | 240 | 368 | 17653 | 2.39850543478261 | 0.00466295079202581 | 0.561792152611332 | 2.33133916761744 | 0.0326086956521739 | PPP3CB//PRKAR2B//CNTN2//ITGA5//ABCC8//VLDLR//ITGA8//CTNS//CPEB3//ACTR2//HIF1A//GRIN2B// |
| GO:0016055 | Wnt signaling pathway | Biological process | 20 | 504 | 368 | 17653 | 1.90357574189096 | 0.00469534392596817 | 0.561792152611332 | 2.32833259089954 | 0.0543478260869565 | FZD2//GNAO1//PPP3CA//PPP3CB//FZD3//FOXP3//SFRP1//TRABD2B//CXXC4//LRRK2//NDP//DIXDC1//PSMD7//CTNND2//DLX5//TNKS2//FERMT2//MARK1//PRKAA1//SKP1// |
| GO:0016043 | cellular component organization | Biological process | 155 | 6269 | 368 | 17653 | 1.18605309424567 | 0.0047182428088294 | 0.561792152611332 | 2.32621971333991 | 0.421195652173913 | UBXN2B//UBXN2A//TP53INP2//BUB3//SEPT7//KIF2A//MARK1//MAP7D1//CNTN2//PAXIP1//SHQ1//COPS5//COPS2//RHOH//AGTR1//HTRA4//EMP2//PLLP//PMAIP1//LRRTM1//RHOF//MICAL2//NISCH//ARFIP2//MAEA//PALM//PFDN1//BFSP1//NDP//SYT14//VAMP3//PURA//RSBN1//ASF1B//HMGB3//FOXP3//ACTL6A//TIMM23//TOMM34//LRRK2//EPAS1//NOS3//BRK1//CGNL1//TLN1//BAG5//PARD6B//ITGA5//TTK//MAPRE1//NUP214//CDKN1B//MCMDC2//CHRNA3//SMAD1//LRRC38//RTN4//EFNA3//NCAM1//NRAS//VLDLR//UNC5A//TNFRSF8//PCDHB3//BSN//P2RX2//FERMT2//PARVA//ACTR3//ACTR2//UBXN10//MCIDAS//SKP1//UBE2E1//SFRP1//ZNF804A//PEX5//ZFP64//NFS1//FBXO45//ITGA8//ARHGEF6//STON2//TOMM40L//JAM3//EIF4G2//GDF9//BMP10//EPS8L2//PARG//PAXBP1//NREP//TRDN//UBE4B//GNAO1//PRDM12//TMEM108//TBC1D22B//NEK7//TNKS2//TRABD2B//DIXDC1//FERMT3//CADM2//HIPK1//DNAJB12//KMT2E//PRKAA1//OTOR//CCDC103//FZD3//GPR3//KCTD18//ALAD//OGT//PRDM4//ERVFRD-1//KRT2//KCNIP2//PPP3CA//CEP85//GOSR2//PPP3CB//SEMA4G//SKIL//TLX2//CTNND2//CTH//DPYS//ACADL//GRIN2B//YWHAB//PAX5//NPTX1//CPEB3//YWHAZ//ANO3//PPIH//MRPS33//MRPL50//ATP6V1G2//DLX5//PRKAR2B//GRIPAP1//SH3YL1//SLC25A5//CALCOCO2//IL4R//TNFSF14//ZSWIM6//HIF1A//UBE2D3//FZD2//ABCC8//PIP4K2C// |
| GO:0033043 | regulation of organelle organization | Biological process | 40 | 1253 | 368 | 17653 | 1.53136819459384 | 0.00472646844179238 | 0.561792152611332 | 2.3254632375061 | 0.108695652173913 | PAXIP1//TTK//BUB3//CDKN1B//BRK1//EPS8L2//RHOH//PARG//ZFP64//PAXBP1//FOXP3//MAPRE1//TBC1D22B//NEK7//TNKS2//RHOF//DIXDC1//ACTR3//ACTR2//ARFIP2//GPR3//CEP85//SFRP1//PAX5//PRDM12//BMP10//OGT//JAM3//LRRK2//PMAIP1//YWHAB//YWHAZ//SLC25A5//CALCOCO2//SEPT7//HIF1A//PRKAA1//UBE2D3//KMT2E//PIP4K2C// |
| GO:0050808 | synapse organization | Biological process | 14 | 304 | 368 | 17653 | 2.20914974256293 | 0.00484988807697944 | 0.561792152611332 | 2.31426828368868 | 0.0380434782608696 | PCDHB3//BSN//LRRK2//P2RX2//LRRTM1//PALM//FBXO45//CTNND2//ACTR2//CNTN2//TMEM108//GRIN2B//ZNF804A//GRIPAP1// |
| GO:0198738 | cell-cell signaling by wnt | Biological process | 20 | 506 | 368 | 17653 | 1.89605172710088 | 0.00490232336069262 | 0.561792152611332 | 2.30959804577003 | 0.0543478260869565 | FERMT2//CTNND2//TRABD2B//MARK1//NDP//PRKAA1//SKP1//CXXC4//TNKS2//FZD2//GNAO1//PPP3CA//PPP3CB//FZD3//FOXP3//SFRP1//LRRK2//DIXDC1//PSMD7//DLX5// |
| GO:0071380 | cellular response to prostaglandin E stimulus | Biological process | 3 | 17 | 368 | 17653 | 8.46531329923274 | 0.00491718399111143 | 0.561792152611332 | 2.30828354098187 | 0.00815217391304348 | PRKAA1//SFRP1//TNFSF4// |
| GO:1902043 | positive regulation of extrinsic apoptotic signaling pathway via death domain receptors | Biological process | 3 | 17 | 368 | 17653 | 8.46531329923274 | 0.00491718399111143 | 0.561792152611332 | 2.30828354098187 | 0.00815217391304348 | PMAIP1//SFRP1//SKIL// |
| GO:0022008 | neurogenesis | Biological process | 47 | 1536 | 368 | 17653 | 1.46783535722373 | 0.00498410899847404 | 0.561792152611332 | 2.30241246857236 | 0.127717391304348 | FBXO45//MARK1//FZD3//OTP//HIF1A//LRRC38//RTN4//PARD6B//EFNA3//NCAM1//NRAS//CNTN2//VLDLR//UNC5A//TNFRSF8//SFRP1//ZNF804A//LRRK2//BAG5//DLX5//HIPK1//FZD2//ALDH1A2//COPS2//JAM3//NREP//UBE4B//GNAO1//PRDM12//TMEM108//KCNIP2//DIXDC1//HEYL//VWC2//SKIL//PPP3CB//RHOH//CHRNA3//SEMA4G//TLX2//PPP3CA//ABCC8//NPTX1//CTNND2//CPEB3//ACTR2//ZSWIM6// |
| GO:0034976 | response to endoplasmic reticulum stress | Biological process | 13 | 274 | 368 | 17653 | 2.27595406220248 | 0.00511145384421633 | 0.566701022105492 | 2.29145555647614 | 0.0353260869565217 | UBE4B//CTH//CREB3L3//KDELR3//TLN1//GOSR2//DNAJB12//LRRK2//PMAIP1//COPS5//PDIA3//NRBF2//TXNDC5// |
| GO:0022029 | telencephalon cell migration | Biological process | 5 | 54 | 368 | 17653 | 4.44167673107891 | 0.00524715487966322 | 0.572363039534877 | 2.28007611659252 | 0.0135869565217391 | FBXO45//DIXDC1//RTN4//CNTN2//LRRK2// |
| GO:0007167 | enzyme linked receptor protein signaling pathway | Biological process | 34 | 1028 | 368 | 17653 | 1.58656001522585 | 0.00533575059711793 | 0.572788591877914 | 2.27280447834691 | 0.0923913043478261 | PTPRT//CD8A//CHRNA3//FAM83B//NRAS//CAMLG//FERMT2//GDF9//SMAD1//SKIL//ATP6V1G2//POLR2E//PTBP1//BMP10//TTK//NREP//DLX5//UBE2D3//ITGA8//HTRA4//RASL11B//VWC2//SFRP1//HIF1A//ITGA5//PTPN18//OGT//BRK1//ACTR3//ACTR2//EFNA3//GRIN2B//TMEM108//RTN4// |
| GO:0051193 | regulation of cofactor metabolic process | Biological process | 7 | 102 | 368 | 17653 | 3.29206628303495 | 0.00548982550445595 | 0.580120154478681 | 2.26044145949458 | 0.0190217391304348 | NUP214//OGT//DLAT//CTNS//HIF1A//PRKAA1//ME2// |
| GO:0050890 | cognition | Biological process | 13 | 277 | 368 | 17653 | 2.25130474022916 | 0.00558967915062426 | 0.58158461685649 | 2.25261312004068 | 0.0353260869565217 | GRIN2B//PPP3CB//PRKAR2B//CNTN2//ITGA5//ABCC8//VLDLR//ITGA8//CTNS//CPEB3//ACTR2//HIF1A//CHRM1// |
| GO:0046639 | negative regulation of alpha-beta T cell differentiation | Biological process | 3 | 18 | 368 | 17653 | 7.99501811594203 | 0.00581042292927985 | 0.583604396437352 | 2.23579225501628 | 0.00815217391304348 | IL4R//TNFSF4//FOXP3// |
| GO:0033044 | regulation of chromosome organization | Biological process | 14 | 311 | 368 | 17653 | 2.15942611491682 | 0.00590056818935116 | 0.583604396437352 | 2.22910616638859 | 0.0380434782608696 | PAXIP1//TTK//BUB3//PARG//ZFP64//PAXBP1//FOXP3//NEK7//TNKS2//PAX5//PRDM12//OGT//LRRK2//KMT2E// |
| GO:0007165 | signal transduction | Biological process | 153 | 6210 | 368 | 17653 | 1.18187224322621 | 0.00590910373769043 | 0.583604396437352 | 2.22847838566578 | 0.415760869565217 | LRRK2//GRIN2B//SMAD1//NCAM1//NRAS//MAPK4//PSMD7//YWHAB//PRKAA1//PROK1//CD81//PMAIP1//SKP1//UBE2D3//SKAP1//FERMT2//CTNND2//TRABD2B//MARK1//NDP//CXXC4//TNKS2//FZD2//GNAO1//PPP3CA//PPP3CB//FZD3//PTPRT//CD8A//CARHSP1//PRKAR2B//POLR2M//SH3BP5//OXSR1//CDKN1B//CLEC1A//TSPAN14//TSPAN18//CHRNA3//FAM83B//CAMLG//GDF9//SKIL//CHRM1//AGTR1//GPR160//GNAZ//GPR34//RIC8B//CXCL11//SFRP1//ARHGEF6//P2RY14//GPR3//PALM//HEYL//HIPK1//EVC//ITGA5//TLN1//FERMT3//ITGA8//DDAH1//NOS3//RALBP1//ARFIP2//RHOH//RHOF//RALGPS2//RRAS//EPS8L2//ATP6V1G2//POLR2E//PTBP1//CD46//UBE4B//PPP1R2//EBI3//FGF11//BMP10//IFNK//TG//TNFSF4//TNFSF14//TTK//PIP4K2C//MGRN1//NISCH//BRK1//NREP//HIF1A//LRRTM1//IL4R//YWHAZ//TNFAIP8L3//FOXP3//SMPD1//PAXIP1//DLX5//HTRA4//RASL11B//VWC2//CTH//CREB3L3//LARP1//RABL3//INSIG2//TNFRSF8//WDFY1//OGT//P2RX2//KDELR3//GOSR2//UNC5A//VLDLR//ACTR3//ACTR2//PTPN18//PAX8//NDFIP1//RNF149//COPS5//EFNA3//ALDH1A2//PAX5//FAM13A//ARHGAP36//CGNL1//TMEM108//RTN4//DIXDC1//CNTN2//JPH1//TRDN//SEMA4G//CLDN18//BSN//LYPD6//SLC25A5//BAG5//LDOC1//EMP2//PDIA3//PRDM4//AKAP10//EPAS1//KCNIP3//KCNIP2//IGSF1//MRC1//ABCC8//ACTL6A//COPS2// |
| GO:0023052 | signaling | Biological process | 164 | 6724 | 368 | 17653 | 1.17000265111347 | 0.00600280233527062 | 0.583604396437352 | 2.22164595717962 | 0.445652173913043 | LRRK2//GRIN2B//SMAD1//NCAM1//NRAS//MAPK4//PSMD7//YWHAB//PRKAA1//PROK1//CD81//PMAIP1//VAMP3//AGTR1//SKP1//UBE2D3//SKAP1//EFNA3//FGF11//NDP//PCDHB3//CXCL11//FERMT2//CTNND2//TRABD2B//MARK1//CXXC4//TNKS2//FZD2//GNAO1//PPP3CA//PPP3CB//FZD3//FAM13A//RALBP1//PRDM4//PTPRT//AKAP10//CHRM1//CHRNA3//ARHGAP36//EPAS1//PDIA3//KCNIP3//KCNIP2//HIF1A//IGSF1//IL4R//MRC1//RASL11B//SMPD1//ABCC8//TG//TNFSF4//VLDLR//YWHAZ//NDFIP1//CAMLG//OGT//ACTL6A//TNFSF14//COPS2//TNFRSF8//SH3BP5//CD8A//CARHSP1//PRKAR2B//POLR2M//OXSR1//CDKN1B//CLEC1A//TSPAN14//TSPAN18//FAM83B//GDF9//SKIL//GPR160//GNAZ//GPR34//RIC8B//SFRP1//ARHGEF6//P2RY14//GPR3//PALM//HEYL//HIPK1//EVC//ITGA5//TLN1//FERMT3//ITGA8//DDAH1//NOS3//ARFIP2//RHOH//RHOF//RALGPS2//RRAS//EPS8L2//CPLX1//NOVA1//NPTX1//BSN//P2RX2//SLC25A5//ATP6V1G2//POLR2E//PTBP1//CD46//UBE4B//PPP1R2//EBI3//BMP10//IFNK//TTK//PIP4K2C//SYT14//MGRN1//NISCH//BRK1//NREP//LRRTM1//JAM3//SCN1A//SCN7A//TNFAIP8L3//FOXP3//PAXIP1//DLX5//HTRA4//VWC2//CTH//CREB3L3//LARP1//RABL3//INSIG2//WDFY1//KDELR3//GOSR2//UNC5A//ACTR3//ACTR2//PTPN18//PAX8//RNF149//COPS5//CPEB3//CNTN2//ALDH1A2//PAX5//CGNL1//TMEM108//RTN4//DIXDC1//JPH1//TRDN//SEMA4G//CLDN18//SCN3B//LYPD6//BAG5//LDOC1//EMP2// |
| GO:0002637 | regulation of immunoglobulin production | Biological process | 5 | 56 | 368 | 17653 | 4.28304541925466 | 0.00612758957976521 | 0.583604396437352 | 2.21271033104066 | 0.0135869565217391 | IL4R//PAXIP1//FOXP3//NDFIP1//TNFSF4// |
| GO:0065008 | regulation of biological quality | Biological process | 99 | 3770 | 368 | 17653 | 1.25969250951447 | 0.00618145256239588 | 0.583604396437352 | 2.20890945926269 | 0.269021739130435 | USP53//ACADL//ABCC8//JAM3//FOXP3//PMAIP1//TP53INP2//HIF1A//AGTR1//EPAS1//SKIL//P2RX2//CHRM1//NOS3//DDAH1//PRKAR2B//CYP4F2//EMP2//AKAP10//PDIA3//TXNDC5//SH3BGRL3//KDELR3//TRDN//LETM2//SKP1//NDFIP1//ATP1B4//TLN1//CPLX1//SLC25A5//PPP3CB//BRK1//NISCH//FERMT2//PALM//RHOF//PARVA//SCN3B//STARD4//MEST//ABHD5//CTNS//CHRNA3//SYT14//SEPT7//RTN4//SCN1A//SCN7A//DPYS//UPB1//YWHAZ//KMT2E//EPS8L2//RHOH//CNTN2//RNF149//CD81//FBXL3//NEK7//TNKS2//ACTR3//ACTR2//ARFIP2//LRRK2//SLC26A1//TG//ALDH1A2//SMAD1//PRKAA1//TNFSF14//MAEA//CARHSP1//PSMD7//YWHAB//NUP214//CLDN18//STATH//PPP3CA//SFRP1//CPEB3//GRIN2B//LARP1//SEMA4G//CXCL11//SLC35G1//POLR2M//LRRTM1//PNPLA5//JPH1//ANO3//FERMT3//KCNIP2//ATP6V1G2//TMEM108//GRIPAP1//ZNF804A//PAX8//GDF9// |
| GO:2001252 | positive regulation of chromosome organization | Biological process | 9 | 159 | 368 | 17653 | 2.71528917145201 | 0.00621314750014629 | 0.583604396437352 | 2.20668833611178 | 0.0244565217391304 | PAXIP1//PAXBP1//NEK7//TNKS2//FOXP3//PRDM12//OGT//LRRK2//KMT2E// |
| GO:0010508 | positive regulation of autophagy | Biological process | 7 | 105 | 368 | 17653 | 3.19800724637681 | 0.00641916179324392 | 0.585228241788634 | 2.19252167790234 | 0.0190217391304348 | LARP1//HIF1A//CALCOCO2//PIP4K2C//LRRK2//PRKAA1//TP53INP2// |
| GO:0034314 | Arp2/3 complex-mediated actin nucleation | Biological process | 4 | 36 | 368 | 17653 | 5.33001207729469 | 0.0064647795544804 | 0.585228241788634 | 2.18944627974028 | 0.0108695652173913 | ARFIP2//BRK1//ACTR3//ACTR2// |
| GO:0016571 | histone methylation | Biological process | 8 | 132 | 368 | 17653 | 2.90727931488801 | 0.00649003668995232 | 0.585228241788634 | 2.18775284800721 | 0.0217391304347826 | PAXIP1//ZFP64//PAXBP1//KMT2E//PRDM4//PAX5//PRDM12//OGT// |
| GO:0021885 | forebrain cell migration | Biological process | 5 | 57 | 368 | 17653 | 4.20790427154844 | 0.00660457745206058 | 0.587720490898496 | 2.18015496247165 | 0.0135869565217391 | FBXO45//DIXDC1//RTN4//CNTN2//LRRK2// |
| GO:0033622 | integrin activation | Biological process | 3 | 19 | 368 | 17653 | 7.57422768878719 | 0.0067945179686867 | 0.589443094613296 | 2.16784134849221 | 0.00815217391304348 | FERMT2//TLN1//FERMT3// |
| GO:0007154 | cell communication | Biological process | 164 | 6744 | 368 | 17653 | 1.16653289236165 | 0.00682580177917923 | 0.589443094613296 | 2.16584632775656 | 0.445652173913043 | LRRK2//GRIN2B//SMAD1//NCAM1//NRAS//MAPK4//PSMD7//YWHAB//PRKAA1//PROK1//CD81//PMAIP1//AGTR1//SKP1//UBE2D3//SKAP1//EFNA3//FGF11//NDP//PCDHB3//CXCL11//FERMT2//CTNND2//TRABD2B//MARK1//CXXC4//TNKS2//FZD2//GNAO1//PPP3CA//PPP3CB//FZD3//FAM13A//RALBP1//PRDM4//PTPRT//AKAP10//CHRM1//CHRNA3//ARHGAP36//EPAS1//PDIA3//KCNIP3//KCNIP2//HIF1A//IGSF1//IL4R//MRC1//RASL11B//SMPD1//ABCC8//TG//TNFSF4//VLDLR//YWHAZ//NDFIP1//CAMLG//OGT//ACTL6A//TNFSF14//COPS2//TNFRSF8//SH3BP5//CD8A//CARHSP1//PRKAR2B//POLR2M//OXSR1//CDKN1B//CLEC1A//TSPAN14//TSPAN18//FAM83B//GDF9//SKIL//GPR160//GNAZ//GPR34//RIC8B//SFRP1//ARHGEF6//P2RY14//GPR3//PALM//HEYL//HIPK1//EVC//ITGA5//TLN1//FERMT3//ITGA8//DDAH1//NOS3//ARFIP2//RHOH//RHOF//RALGPS2//RRAS//EPS8L2//CPLX1//NOVA1//NPTX1//BSN//P2RX2//SLC25A5//ATP6V1G2//POLR2E//PTBP1//CD46//UBE4B//PPP1R2//EBI3//BMP10//IFNK//TRDN//TTK//PIP4K2C//SYT14//MGRN1//NISCH//BRK1//NREP//LRRTM1//JAM3//SCN1A//SCN7A//TNFAIP8L3//FOXP3//PAXIP1//DLX5//HTRA4//VWC2//CTH//CREB3L3//LARP1//RABL3//INSIG2//WDFY1//KDELR3//GOSR2//UNC5A//ACTR3//ACTR2//PTPN18//PAX8//NUDT15//RNF149//COPS5//CPEB3//CNTN2//ALDH1A2//PAX5//CGNL1//TMEM108//RTN4//DIXDC1//JPH1//SEMA4G//CLDN18//SCN3B//LYPD6//BAG5//LDOC1//EMP2// |
| GO:0002456 | T cell mediated immunity | Biological process | 6 | 81 | 368 | 17653 | 3.55334138486312 | 0.00688540654656963 | 0.589443094613296 | 2.16207041187485 | 0.016304347826087 | EMP2//PPP3CB//FOXP3//TNFSF4//CD46//CD8A// |
| GO:0065007 | biological regulation | Biological process | 279 | 12351 | 368 | 17653 | 1.08360945073977 | 0.00701016412269408 | 0.592621749522251 | 2.15427181416658 | 0.758152173913043 | CDKN1B//CPEB3//HEYL//KCNIP3//PAX5//FOXP3//RLIM//SKIL//UBE2D3//VLDLR//BHLHE40//COPS2//PAXBP1//LRRK2//GRIN2B//SMAD1//NCAM1//NRAS//MAPK4//PSMD7//YWHAB//PNRC1//PRKAA1//PROK1//CD81//EIF4A2//RBFOX1//PTBP1//PAXIP1//USP53//AGTR1//HTRA4//ACADL//ABCC8//JAM3//TNFSF4//PMAIP1//CAMLG//TP53INP2//PPP3CB//VAMP3//HIF1A//ALDH1A2//EMP2//SFRP1//EPAS1//NOS3//OTP//FZD3//LRRTM1//SKP1//IL4R//CREB3L3//CXCL11//SKAP1//NDFIP1//P2RX2//CHRM1//DDAH1//PRKAR2B//CYP4F2//COPS5//NUP214//AKAP10//FERMT2//CTNND2//TRABD2B//MARK1//NDP//CXXC4//TNKS2//FZD2//GNAO1//PPP3CA//FAM13A//RALBP1//PRDM4//PTPRT//CHRNA3//ARHGAP36//FGF11//PDIA3//KCNIP2//IGSF1//MRC1//RASL11B//SMPD1//TG//YWHAZ//OGT//ACTL6A//TNFSF14//TNFRSF8//SH3BP5//CD8A//CARHSP1//POLR2M//OXSR1//PPP1R3F//TXNDC5//SH3BGRL3//MCIDAS//PARG//KRBOX1//ZBTB6//ZNF618//HIPK1//EWSR1//ZBTB43//ATP1B4//MYCBP//NRBF2//HMGB3//RHOH//MLF1//UCHL5//PFDN1//ASF1B//ZFP64//IFNK//ZNF124//ZNF322//ELK1//FOXG1//HOXC9//PURA//CREBZF//PRDM12//SIM1//VEZF1//CREB5//RBM3//EIF4G2//CEP85//KDELR3//TRDN//LETM2//CD46//TLN1//KMT2E//TTK//BUB3//CLEC1A//TSPAN14//TSPAN18//FAM83B//GDF9//GPR160//GNAZ//GPR34//RIC8B//ARHGEF6//P2RY14//GPR3//PALM//EVC//ITGA5//FERMT3//ITGA8//ARFIP2//RHOF//RALGPS2//RRAS//EPS8L2//CPLX1//MAEA//NEK7//TTC28//SLC25A5//BRK1//NISCH//PGGT1B//C18ORF54//LDOC1//DEC1//ATP6V1G2//PARVA//NUDT15//POLR2E//UBE4B//CASP7//PYURF//PPP1R2//MAPRE1//SCN3B//UBXN2A//EBI3//BMP10//DRAM1//PIP4K2C//DLAT//ZNF804A//CAPN7//RTN4//PAX8//CTNS//MICAL2//VWC2//STARD4//MEST//ABHD5//INSIG2//RRP1B//ZCCHC9//PPP1R26//A2ML1//CNTN2//BAG5//SYT14//LARP1//MGRN1//TNFAIP8L3//SEPT7//EFNA3//DLX5//NREP//MEX3B//SCN1A//SCN7A//DPYS//UPB1//STON2//KIF2A//BEX4//PARD6B//SEMA4G//STATH//CTH//TBC1D22B//RNF149//FBXL3//RNF217//RNF135//RABL3//DIXDC1//RFK//WDFY1//ACTR3//ACTR2//KCNQ4//GOSR2//UNC5A//PTPN18//SLC26A1//SHQ1//WDR91//SLC5A3//UBXN2B//PPIH//IDI1//CLDN18//TLX2//TMPRSS2//BAG2//CGNL1//NOVA1//TMEM108//SLC35G1//BEST1//PNPLA5//JPH1//ANO3//GRIPAP1//BSN//LYPD6//SH3YL1//LSM11//SNRPA//PEX5//CALCOCO2//ALAD//ME2//AOC3//ZSWIM6//NUCB1//LRRC38//SESTD1//FAM168A//RNF169// |
| GO:0009653 | anatomical structure morphogenesis | Biological process | 73 | 2656 | 368 | 17653 | 1.31845554773442 | 0.007192332306594 | 0.600515350487595 | 2.14313025514774 | 0.198369565217391 | ERVFRD-1//RHOH//OTOR//EPAS1//HIF1A//ITGA5//NOS3//RTN4//VEZF1//JAM3//PROK1//PAXIP1//PAX8//TLX2//SMAD1//FZD3//SKIL//PPP3CB//CCDC103//MICAL2//DLX5//PARVA//ALDH1A2//FZD2//HEYL//UBE4B//PAX5//LRRC38//PARD6B//EFNA3//NCAM1//NRAS//CNTN2//VLDLR//UNC5A//TNFRSF8//FERMT2//PALM//RHOF//HIPK1//SFRP1//SEPT7//ABCC8//FBXO45//FERMT3//ITGA8//VAMP3//LRRK2//INSIG2//KCNQ4//BMP10//EMP2//DDAH1//RRAS//C2ORF49//HOXC9//CHRNA3//SEMA4G//UCHL5//PPP3CA//PSMD7//CRISPLD1//NPTX1//CTNND2//ACTR2//NDP//TMEM108//IL4R//TNFSF14//ZSWIM6//AGTR1//FOXG1//FOXP3// |
| GO:0051960 | regulation of nervous system development | Biological process | 28 | 820 | 368 | 17653 | 1.63800371155885 | 0.00735065977005405 | 0.600840799020437 | 2.13367367838053 | 0.0760869565217391 | OTP//HIF1A//FZD3//SFRP1//ZNF804A//LRRK2//TNFRSF8//BAG5//RTN4//CNTN2//TG//EFNA3//NREP//DIXDC1//HEYL//VWC2//CHRNA3//SEMA4G//SKIL//TLX2//PPP3CA//LRRTM1//ABCC8//CPEB3//ACTR2//FZD2//VLDLR//ZSWIM6// |
| GO:0007409 | axonogenesis | Biological process | 18 | 456 | 368 | 17653 | 1.8935569221968 | 0.00750627242413546 | 0.600840799020437 | 2.12457567784569 | 0.0489130434782609 | EFNA3//NCAM1//NRAS//CNTN2//VLDLR//UNC5A//TNFRSF8//FBXO45//RTN4//FZD3//PPP3CB//SEMA4G//SKIL//NPTX1//DLX5//ZSWIM6//LRRC38//PARD6B// |
| GO:0048562 | embryonic organ morphogenesis | Biological process | 13 | 288 | 368 | 17653 | 2.16531740640097 | 0.0076575476204386 | 0.600840799020437 | 2.11591029374053 | 0.0353260869565217 | HIF1A//CCDC103//MICAL2//DLX5//INSIG2//PAX8//FZD3//ITGA8//KCNQ4//HIPK1//PAX5//HOXC9//FZD2// |
| GO:0016601 | Rac protein signal transduction | Biological process | 4 | 38 | 368 | 17653 | 5.04948512585812 | 0.00784195559568273 | 0.600840799020437 | 2.10557562117947 | 0.0108695652173913 | OGT//NISCH//RHOH//BRK1// |
| GO:0071840 | cellular component organization or biogenesis | Biological process | 158 | 6486 | 368 | 17653 | 1.1685595396104 | 0.00784396277605632 | 0.600840799020437 | 2.10546447596699 | 0.429347826086957 | UBXN2B//UBXN2A//TP53INP2//BUB3//SEPT7//THUMPD1//KIF2A//MARK1//MAP7D1//CNTN2//PAXIP1//SHQ1//COPS5//COPS2//RHOH//AGTR1//HTRA4//EMP2//PLLP//PMAIP1//LRRTM1//RHOF//MICAL2//NISCH//ARFIP2//MAEA//PALM//PFDN1//BFSP1//NDP//SYT14//VAMP3//PURA//RSBN1//ASF1B//HMGB3//FOXP3//ACTL6A//RPP14//RRP1B//TIMM23//TOMM34//LRRK2//EPAS1//NOS3//BRK1//CGNL1//TLN1//BAG5//PARD6B//ITGA5//TTK//MAPRE1//NUP214//CDKN1B//MCMDC2//CHRNA3//SMAD1//LRRC38//RTN4//EFNA3//NCAM1//NRAS//VLDLR//UNC5A//TNFRSF8//PCDHB3//BSN//P2RX2//FERMT2//PARVA//ACTR3//ACTR2//UBXN10//MCIDAS//SKP1//UBE2E1//SFRP1//ZNF804A//PEX5//ZFP64//NFS1//FBXO45//ITGA8//ARHGEF6//STON2//TOMM40L//JAM3//EIF4G2//GDF9//BMP10//EPS8L2//PARG//PAXBP1//NREP//TRDN//UBE4B//GNAO1//PRDM12//TMEM108//TBC1D22B//NEK7//TNKS2//TRABD2B//DIXDC1//FERMT3//CADM2//HIPK1//DNAJB12//KMT2E//PRKAA1//OTOR//CCDC103//FZD3//GPR3//KCTD18//ALAD//OGT//PRDM4//ERVFRD-1//KRT2//KCNIP2//PPP3CA//CEP85//GOSR2//PPP3CB//SEMA4G//SKIL//TLX2//CTNND2//CTH//DPYS//ACADL//GRIN2B//YWHAB//PAX5//NPTX1//CPEB3//YWHAZ//ANO3//PPIH//MRPS33//MRPL50//ATP6V1G2//DLX5//PRKAR2B//GRIPAP1//SH3YL1//SLC25A5//CALCOCO2//IL4R//TNFSF14//ZSWIM6//HIF1A//UBE2D3//FZD2//ABCC8//PIP4K2C// |
| GO:0002052 | positive regulation of neuroblast proliferation | Biological process | 3 | 20 | 368 | 17653 | 7.19551630434783 | 0.00787162305136396 | 0.600840799020437 | 2.10393571115093 | 0.00815217391304348 | OTP//HIF1A//FZD3// |
| GO:2000515 | negative regulation of CD4-positive, alpha-beta T cell activation | Biological process | 3 | 20 | 368 | 17653 | 7.19551630434783 | 0.00787162305136396 | 0.600840799020437 | 2.10393571115093 | 0.00815217391304348 | IL4R//TNFSF4//FOXP3// |
| GO:0006457 | protein folding | Biological process | 12 | 257 | 368 | 17653 | 2.23984943326002 | 0.00790879132793654 | 0.600840799020437 | 2.10188988309099 | 0.0326086956521739 | HSPA13//PDIA3//DNAJB12//BAG5//PPIH//TOMM34//GNAO1//GNAZ//PFDN1//RPAP3//TXNDC5//BAG2// |
| GO:0120035 | regulation of plasma membrane bounded cell projection organization | Biological process | 22 | 602 | 368 | 17653 | 1.75306045067167 | 0.00799581131329873 | 0.600840799020437 | 2.09713746299815 | 0.0597826086956522 | BRK1//SFRP1//ZNF804A//LRRK2//TNFRSF8//BAG5//RTN4//CNTN2//CHRNA3//SEMA4G//SKIL//TLX2//PPP3CA//PALM//CPEB3//ACTR2//VLDLR//SH3YL1//EPS8L2//ZSWIM6//SEPT7//GRIN2B// |
| GO:0007613 | memory | Biological process | 7 | 110 | 368 | 17653 | 3.05264328063241 | 0.0082215587726403 | 0.611015406366663 | 2.08504583426321 | 0.0190217391304348 | CTNS//CPEB3//ITGA5//PPP3CB//ABCC8//VLDLR//ITGA8// |
| GO:0032989 | cellular component morphogenesis | Biological process | 35 | 1101 | 368 | 17653 | 1.52493533546578 | 0.00857226948818216 | 0.619471109154853 | 2.0669041844115 | 0.0951086956521739 | RHOH//LRRC38//RTN4//PARD6B//EFNA3//NCAM1//NRAS//CNTN2//VLDLR//UNC5A//TNFRSF8//FERMT2//PALM//RHOF//PARVA//SEPT7//FBXO45//FERMT3//ITGA8//VAMP3//FZD3//BMP10//PPP3CB//LRRK2//CHRNA3//SEMA4G//SKIL//TLX2//PPP3CA//NPTX1//CTNND2//ACTR2//DLX5//TMEM108//ZSWIM6// |
| GO:0043029 | T cell homeostasis | Biological process | 4 | 39 | 368 | 17653 | 4.92001114827202 | 0.00859742004376232 | 0.619471109154853 | 2.06563185443753 | 0.0108695652173913 | FOXP3//PMAIP1//PPP3CB//TNFSF14// |
| GO:0050794 | regulation of cellular process | Biological process | 253 | 11074 | 368 | 17653 | 1.09593981397869 | 0.00861012631384832 | 0.619471109154853 | 2.06499047723335 | 0.6875 | CDKN1B//CPEB3//HEYL//KCNIP3//PAX5//FOXP3//RLIM//SKIL//UBE2D3//VLDLR//BHLHE40//COPS2//PAXBP1//LRRK2//GRIN2B//SMAD1//NCAM1//NRAS//MAPK4//PSMD7//YWHAB//PRKAA1//PROK1//CD81//RBFOX1//PTBP1//PAXIP1//AGTR1//HTRA4//PMAIP1//VAMP3//ALDH1A2//HIF1A//OTP//FZD3//LRRTM1//SKP1//CXCL11//SKAP1//NDFIP1//COPS5//NUP214//FERMT2//CTNND2//TRABD2B//MARK1//NDP//CXXC4//TNKS2//FZD2//GNAO1//PPP3CA//PPP3CB//FAM13A//RALBP1//PRDM4//PTPRT//AKAP10//CHRM1//CHRNA3//ARHGAP36//EPAS1//FGF11//PDIA3//KCNIP2//IGSF1//IL4R//MRC1//RASL11B//SMPD1//ABCC8//TG//TNFSF4//YWHAZ//CAMLG//OGT//ACTL6A//TNFSF14//TNFRSF8//SH3BP5//CD8A//CARHSP1//PRKAR2B//POLR2M//OXSR1//PPP1R3F//NOS3//TXNDC5//SH3BGRL3//MCIDAS//PARG//KRBOX1//ZBTB6//PNRC1//ZNF618//HIPK1//EWSR1//ZBTB43//ATP1B4//MYCBP//NRBF2//HMGB3//RHOH//MLF1//UCHL5//PFDN1//ASF1B//ZFP64//IFNK//ZNF124//ZNF322//ELK1//FOXG1//HOXC9//PURA//CREBZF//PRDM12//SIM1//VEZF1//CREB5//RBM3//EIF4A2//EIF4G2//CEP85//KMT2E//TTK//BUB3//CLEC1A//TSPAN14//TSPAN18//FAM83B//GDF9//GPR160//GNAZ//GPR34//RIC8B//SFRP1//ARHGEF6//P2RY14//GPR3//PALM//EVC//ITGA5//TLN1//FERMT3//ITGA8//DDAH1//ARFIP2//RHOF//RALGPS2//RRAS//EPS8L2//MAEA//NEK7//TTC28//SLC25A5//BRK1//EMP2//PGGT1B//C18ORF54//LDOC1//DEC1//ATP6V1G2//PARVA//NUDT15//POLR2E//CD46//UBE4B//CASP7//PPP1R2//MAPRE1//EBI3//BMP10//DRAM1//PIP4K2C//TP53INP2//DLAT//P2RX2//CAPN7//RTN4//TRDN//PAX8//CTNS//MICAL2//STARD4//ABHD5//RRP1B//ZCCHC9//PPP1R26//A2ML1//CNTN2//ZNF804A//BAG5//LARP1//MGRN1//TNFAIP8L3//SEPT7//DLX5//NISCH//NREP//MEX3B//CPLX1//SYT14//STON2//KIF2A//BEX4//PARD6B//SEMA4G//VWC2//CTH//CREB3L3//TBC1D22B//UBXN2A//RNF217//RABL3//INSIG2//DIXDC1//WDFY1//ACTR3//ACTR2//KDELR3//GOSR2//UNC5A//PTPN18//SHQ1//RNF149//WDR91//UBXN2B//EFNA3//ACADL//TLX2//CGNL1//NOVA1//TMEM108//JPH1//SCN3B//CLDN18//JAM3//BSN//LYPD6//SH3YL1//BAG2//LSM11//SNRPA//PEX5//CALCOCO2//ALAD//ME2//ZSWIM6//LRRC38//FAM168A//GRIPAP1//RNF169// |
| GO:0031344 | regulation of cell projection organization | Biological process | 22 | 612 | 368 | 17653 | 1.72441567206593 | 0.00957887445202678 | 0.676188577973527 | 2.01868551889553 | 0.0597826086956522 | BRK1//SFRP1//ZNF804A//LRRK2//TNFRSF8//BAG5//RTN4//CNTN2//CHRNA3//SEMA4G//SKIL//TLX2//PPP3CA//PALM//CPEB3//ACTR2//VLDLR//SH3YL1//EPS8L2//ZSWIM6//SEPT7//GRIN2B// |
| GO:0032446 | protein modification by small protein conjugation | Biological process | 30 | 915 | 368 | 17653 | 1.57279044903778 | 0.00971001280611844 | 0.676188577973527 | 2.0127801973193 | 0.0815217391304348 | UBE4B//RNF217//MGRN1//FBXL3//RLIM//PSMD7//SKP1//UBE2D3//UBE2E1//TNKS2//MAEA//FBXO45//EPAS1//RNF169//RNF149//HIF1A//KLHL7//TULP4//KLHL14//PEX5//TRIM7//RNF135//KBTBD6//NUP214//UBXN2A//OGT//BAG5//LRRK2//PAXIP1//NDFIP1// |
| GO:0019722 | calcium-mediated signaling | Biological process | 9 | 171 | 368 | 17653 | 2.52474256292906 | 0.00980382350787282 | 0.676188577973527 | 2.00860451568259 | 0.0244565217391304 | PPP3CA//PPP3CB//P2RX2//JPH1//TRDN//PRKAA1//LRRK2//AGTR1//GRIN2B// |
| GO:0051196 | regulation of coenzyme metabolic process | Biological process | 6 | 88 | 368 | 17653 | 3.27068922924901 | 0.0101925933500781 | 0.676188577973527 | 1.99171530231994 | 0.016304347826087 | NUP214//OGT//DLAT//HIF1A//PRKAA1//ME2// |
| GO:0035590 | purinergic nucleotide receptor signaling pathway | Biological process | 3 | 22 | 368 | 17653 | 6.54137845849802 | 0.0103121973323273 | 0.676188577973527 | 1.98664878499514 | 0.00815217391304348 | GPR34//P2RY14//P2RX2// |
| GO:0071379 | cellular response to prostaglandin stimulus | Biological process | 3 | 22 | 368 | 17653 | 6.54137845849802 | 0.0103121973323273 | 0.676188577973527 | 1.98664878499514 | 0.00815217391304348 | PRKAA1//SFRP1//TNFSF4// |
| GO:0048856 | anatomical structure development | Biological process | 143 | 5836 | 368 | 17653 | 1.17541561745686 | 0.0104432811371467 | 0.676188577973527 | 1.98116303046411 | 0.388586956521739 | ERVFRD-1//RHOH//CLEC3A//DLX5//EVC//OTOR//EPAS1//HIF1A//ITGA5//NOS3//RTN4//VEZF1//JAM3//PROK1//GDF9//ALDH1A2//PAXIP1//HIPK1//PAX8//ITGA8//SMAD1//SFRP1//SIM1//BRK1//TLX2//FZD3//SEMA4G//FBXO45//MARK1//AGTR1//SKIL//CDKN1B//NDP//LDOC1//PPP3CB//CCDC103//MICAL2//PARVA//OTP//CTNS//STON2//MLF1//FOXP3//RRAS//NDFIP1//FZD2//HEYL//UBE4B//KRT2//ACTL6A//GRIN2B//HMGB3//PAX5//STRBP//ASF1B//TMEFF1//TNFRSF8//FOXG1//CXXC4//CHRM1//CHRNA3//FGF11//KIF2A//RBFOX1//SCN3B//PCDHB3//PURA//SMPD1//VLDLR//LRRC38//PARD6B//EFNA3//NCAM1//NRAS//CNTN2//UNC5A//BSN//NPTX1//CA10//SLC5A3//MEST//BMP10//JPH1//PAXBP1//EMP2//LRRK2//FERMT2//PALM//RHOF//EVPLL//MEA1//HOXC9//ZNF804A//BAG5//SEPT7//ABCC8//TMEM108//UCHL5//DIXDC1//COPS2//IFNK//KMT2E//STATH//TG//GNAO1//NREP//PRDM12//KRTAP1-1//FERMT3//VAMP3//INSIG2//KCNQ4//PLLP//GAL3ST1//GPD2//MAEA//TNFSF4//CD46//SLC27A4//CD8A//KCNIP2//IL4R//TNFSF14//VWC2//DDAH1//BFSP1//MCMDC2//CLDN18//C2ORF49//PRKAA1//P2RX2//C11ORF88//PPP3CA//PTBP1//LRRTM1//PSMD7//CRISPLD1//CTNND2//CPEB3//ACTR2//ZSWIM6//MCIDAS//CTH// |
| GO:0044257 | cellular protein catabolic process | Biological process | 25 | 730 | 368 | 17653 | 1.64281194163192 | 0.0106387609018676 | 0.676188577973527 | 1.9731089514412 | 0.0679347826086956 | UBE4B//RNF217//FBXO45//RLIM//UCHL5//TP53INP2//UBE2D3//UBE2E1//NUDT15//PMAIP1//PSMD7//BUB3//FBXL3//SKP1//OGT//BAG5//LRRK2//VLDLR//DNAJB12//MAEA//UBXN2B//UBXN2A//ALAD//WDR91//CD81// |
| GO:0043161 | proteasome-mediated ubiquitin-dependent protein catabolic process | Biological process | 16 | 403 | 368 | 17653 | 1.90452044449239 | 0.0107893207664097 | 0.676188577973527 | 1.96700589513964 | 0.0434782608695652 | UBE4B//PSMD7//UBE2E1//BUB3//FBXL3//SKP1//OGT//BAG5//LRRK2//RNF217//MAEA//UBXN2B//UBXN2A//FBXO45//RLIM//UBE2D3// |
| GO:0071772 | response to BMP | Biological process | 9 | 174 | 368 | 17653 | 2.48121251874063 | 0.0109065335367629 | 0.676188577973527 | 1.96231326087583 | 0.0244565217391304 | DLX5//GDF9//BMP10//SMAD1//UBE2D3//VWC2//SFRP1//SKIL//HEYL// |
| GO:0071773 | cellular response to BMP stimulus | Biological process | 9 | 174 | 368 | 17653 | 2.48121251874063 | 0.0109065335367629 | 0.676188577973527 | 1.96231326087583 | 0.0244565217391304 | DLX5//GDF9//BMP10//SMAD1//UBE2D3//VWC2//SFRP1//SKIL//HEYL// |
| GO:0050767 | regulation of neurogenesis | Biological process | 25 | 732 | 368 | 17653 | 1.63832338441435 | 0.010982340443732 | 0.676188577973527 | 1.95930509763789 | 0.0679347826086956 | OTP//HIF1A//FZD3//SFRP1//ZNF804A//LRRK2//TNFRSF8//BAG5//RTN4//CNTN2//EFNA3//NREP//DIXDC1//HEYL//VWC2//CHRNA3//SEMA4G//SKIL//TLX2//PPP3CA//ABCC8//CPEB3//ACTR2//VLDLR//ZSWIM6// |
| GO:0007610 | behavior | Biological process | 21 | 583 | 368 | 17653 | 1.72791129092401 | 0.0109918233574901 | 0.676188577973527 | 1.9589302594952 | 0.0570652173913043 | GRIN2B//PPP3CB//PRKAR2B//CNTN2//ITGA5//ABCC8//VLDLR//ITGA8//CTNS//CPEB3//CHRNA3//GNAO1//LRRTM1//NOVA1//SCN1A//STRBP//PAX5//ACTR2//HIF1A//LRRK2//P2RX2// |
| GO:0002204 | somatic recombination of immunoglobulin genes involved in immune response | Biological process | 4 | 42 | 368 | 17653 | 4.5685817805383 | 0.0111441705403195 | 0.676188577973527 | 1.95295225048278 | 0.0108695652173913 | PAXIP1//FOXP3//NDFIP1//TNFSF4// |
| GO:0002208 | somatic diversification of immunoglobulins involved in immune response | Biological process | 4 | 42 | 368 | 17653 | 4.5685817805383 | 0.0111441705403195 | 0.676188577973527 | 1.95295225048278 | 0.0108695652173913 | PAXIP1//FOXP3//NDFIP1//TNFSF4// |
| GO:0045190 | isotype switching | Biological process | 4 | 42 | 368 | 17653 | 4.5685817805383 | 0.0111441705403195 | 0.676188577973527 | 1.95295225048278 | 0.0108695652173913 | PAXIP1//FOXP3//NDFIP1//TNFSF4// |
| GO:0007178 | transmembrane receptor protein serine/threonine kinase signaling pathway | Biological process | 14 | 336 | 368 | 17653 | 1.99875452898551 | 0.0112252497729058 | 0.676188577973527 | 1.94980398673552 | 0.0380434782608696 | FERMT2//GDF9//SMAD1//SKIL//BMP10//TTK//NREP//DLX5//UBE2D3//ITGA8//HTRA4//RASL11B//VWC2//SFRP1// |
| GO:0003073 | regulation of systemic arterial blood pressure | Biological process | 6 | 90 | 368 | 17653 | 3.19800724637681 | 0.011314674819984 | 0.676188577973527 | 1.94635792300982 | 0.016304347826087 | AGTR1//P2RX2//EMP2//CYP4F2//NOS3//DDAH1// |
| GO:0042471 | ear morphogenesis | Biological process | 7 | 117 | 368 | 17653 | 2.87000650315868 | 0.0113369464608192 | 0.676188577973527 | 1.94550390433145 | 0.0190217391304348 | DLX5//INSIG2//PAX8//FZD3//ITGA8//KCNQ4//FZD2// |
| GO:0022603 | regulation of anatomical structure morphogenesis | Biological process | 35 | 1123 | 368 | 17653 | 1.49506126834179 | 0.0113981218230049 | 0.676188577973527 | 1.94316670560258 | 0.0951086956521739 | FERMT2//PALM//RHOF//PARVA//SEPT7//EFNA3//ABCC8//RTN4//CNTN2//EMP2//SFRP1//PROK1//HIPK1//DDAH1//HIF1A//NOS3//RRAS//CHRNA3//SEMA4G//SKIL//TLX2//PPP3CA//FZD2//PSMD7//FZD3//BMP10//LRRK2//ACTR2//PAX8//IL4R//TNFSF14//ZSWIM6//AGTR1//ITGA5//SMAD1// |
| GO:0045940 | positive regulation of steroid metabolic process | Biological process | 3 | 23 | 368 | 17653 | 6.25697069943289 | 0.0116787295658354 | 0.680890069428835 | 1.93260439802465 | 0.00815217391304348 | STARD4//AGTR1//PRKAA1// |
| GO:1901522 | positive regulation of transcription from RNA polymerase II promoter involved in cellular response to chemical stimulus | Biological process | 3 | 23 | 368 | 17653 | 6.25697069943289 | 0.0116787295658354 | 0.680890069428835 | 1.93260439802465 | 0.00815217391304348 | HIF1A//DLX5//SMAD1// |
| GO:0006513 | protein monoubiquitination | Biological process | 5 | 66 | 368 | 17653 | 3.63409914361001 | 0.0121189197701105 | 0.700514994916729 | 1.9165360896407 | 0.0135869565217391 | UBE2E1//SKP1//UBE4B//MGRN1//UBE2D3// |
| GO:1902106 | negative regulation of leukocyte differentiation | Biological process | 6 | 92 | 368 | 17653 | 3.12848534971645 | 0.0125213201239046 | 0.717641423711583 | 1.90234988100551 | 0.016304347826087 | HMGB3//SFRP1//TNFSF4//IL4R//FOXP3//CLDN18// |
| GO:0019932 | second-messenger-mediated signaling | Biological process | 14 | 342 | 368 | 17653 | 1.96368866005594 | 0.0129392384378633 | 0.728653462845222 | 1.88809128409751 | 0.0380434782608696 | GPR3//LRRK2//AGTR1//GRIN2B//DDAH1//NOS3//PPP3CA//PPP3CB//CXCL11//MGRN1//P2RX2//JPH1//TRDN//PRKAA1// |
| GO:0003071 | renal system process involved in regulation of systemic arterial blood pressure | Biological process | 3 | 24 | 368 | 17653 | 5.99626358695652 | 0.0131444214796861 | 0.728653462845222 | 1.88125852357123 | 0.00815217391304348 | AGTR1//EMP2//CYP4F2// |
| GO:0034695 | response to prostaglandin E | Biological process | 3 | 24 | 368 | 17653 | 5.99626358695652 | 0.0131444214796861 | 0.728653462845222 | 1.88125852357123 | 0.00815217391304348 | PRKAA1//SFRP1//TNFSF4// |
| GO:0035588 | G-protein coupled purinergic receptor signaling pathway | Biological process | 3 | 24 | 368 | 17653 | 5.99626358695652 | 0.0131444214796861 | 0.728653462845222 | 1.88125852357123 | 0.00815217391304348 | GPR34//P2RY14//CNTN2// |
| GO:0062012 | regulation of small molecule metabolic process | Biological process | 18 | 485 | 368 | 17653 | 1.78033393097266 | 0.0135260143351721 | 0.743710853242024 | 1.86883015663544 | 0.0489130434782609 | PPP1R3F//NUP214//OGT//PSMD7//PALM//DLAT//PMAIP1//CXCL11//FZD2//NOS3//IDI1//PRKAA1//ACADL//INSIG2//MAEA//HIF1A//STARD4//ME2// |
| GO:2000026 | regulation of multicellular organismal development | Biological process | 56 | 2008 | 368 | 17653 | 1.33781179629309 | 0.0142506189078415 | 0.772174740185072 | 1.84616627373101 | 0.152173913043478 | OTP//HIF1A//FZD3//FOXP3//NDFIP1//SFRP1//ZNF804A//LRRK2//TNFRSF8//BAG5//SEPT7//EFNA3//ABCC8//STATH//RTN4//CNTN2//TG//TNFSF4//CD46//HMGB3//VEZF1//IL4R//KMT2E//NREP//DIXDC1//HEYL//VWC2//EMP2//PROK1//HIPK1//DDAH1//NOS3//RRAS//PAXIP1//PRKAA1//CHRNA3//SEMA4G//SKIL//TLX2//PPP3CA//LRRTM1//BMP10//FZD2//PSMD7//CDKN1B//CPEB3//ACTR2//SMAD1//PAX8//VLDLR//ZSWIM6//AGTR1//ITGA5//CTH//GDF9//CLDN18// |
| GO:0002088 | lens development in camera-type eye | Biological process | 5 | 69 | 368 | 17653 | 3.47609483301827 | 0.0144936101201695 | 0.772174740185072 | 1.83882342543366 | 0.0135869565217391 | HIPK1//SKIL//BFSP1//CDKN1B//CTNS// |
| GO:0023051 | regulation of signaling | Biological process | 92 | 3575 | 368 | 17653 | 1.23447552447552 | 0.0145845649287252 | 0.772174740185072 | 1.83610652176145 | 0.25 | LRRK2//PRKAA1//PROK1//CD81//CAMLG//VAMP3//AGTR1//SLC25A5//PPP3CB//ABCC8//RIC8B//CD46//PPP1R2//SKAP1//EBI3//FGF11//GDF9//BMP10//NDP//IFNK//CXCL11//TG//TNFSF4//TNFSF14//HIPK1//TTK//CHRNA3//PIP4K2C//MGRN1//YWHAB//NREP//SMPD1//FOXP3//SFRP1//TRABD2B//CXXC4//ITGA8//HTRA4//SKIL//RASL11B//VWC2//HIF1A//ITGA5//NISCH//RALGPS2//WDFY1//OGT//EPS8L2//ARHGEF6//CTH//NDFIP1//RHOH//RNF149//PMAIP1//TSPAN14//EVC//COPS5//LRRTM1//PPP3CA//CPLX1//CPEB3//GRIN2B//CNTN2//P2RX2//PAX5//FAM13A//RALBP1//ARHGAP36//RHOF//CGNL1//TMEM108//RRAS//TNFAIP8L3//RTN4//PALM//JPH1//TRDN//HEYL//CTNND2//PSMD7//DLX5//TNKS2//DIXDC1//LYPD6//YWHAZ//PTPN18//NOS3//BAG5//LDOC1//PAX8//EMP2//PDIA3// |
| GO:0043372 | positive regulation of CD4-positive, alpha-beta T cell differentiation | Biological process | 3 | 25 | 368 | 17653 | 5.75641304347826 | 0.0147102528959403 | 0.772174740185072 | 1.83237986089808 | 0.00815217391304348 | FOXP3//IL4R//TNFSF4// |
| GO:0045664 | regulation of neuron differentiation | Biological process | 21 | 600 | 368 | 17653 | 1.67895380434783 | 0.0147636005035125 | 0.772174740185072 | 1.83080771513362 | 0.0570652173913043 | SFRP1//ZNF804A//LRRK2//TNFRSF8//BAG5//RTN4//CNTN2//DIXDC1//HEYL//VWC2//CHRNA3//SEMA4G//SKIL//TLX2//PPP3CA//CPEB3//ACTR2//VLDLR//ZSWIM6//EFNA3//NREP// |
| GO:0051179 | localization | Biological process | 156 | 6497 | 368 | 17653 | 1.15181421525654 | 0.0147740439425652 | 0.772174740185072 | 1.83050061358732 | 0.423913043478261 | PDIA3//SPRN//PPP3CA//NUP214//SLC27A4//HIF1A//SEMA4G//FBXO45//MARK1//FZD3//PLLP//PMAIP1//AGTR1//NOS3//LRRTM1//JAM3//TLN1//FERMT3//CXCL11//HSPA13//VAMP3//AKAP10//MAPRE1//CD81//SYT14//U2AF1//YWHAB//YWHAZ//GOLGA7B//KDELR3//GOSR2//TIMM23//TOMM34//SLC26A1//CHRNA3//CTNS//TOMM40L//P2RX2//CDKN1B//KCNIP2//ABCC8//KCNQ4//SCN3B//SCN1A//SCN7A//SLC5A3//SLC23A2//BEST1//NDFIP1//SLC39A2//SLC25A5//RALBP1//ABCA13//PCTP//ANO3//VLDLR//AP1S1//SNX31//ARFIP2//RABL3//KCNIP3//TBC1D22B//MLPH//CPLX1//OTOR//KIF2A//SCAMP1//SYS1//LRRK2//RHOH//MRC1//TMEM108//TMPRSS2//VPS53//PPP3CB//MGRN1//SLC35A2//ATP1B4//EMP2//BMP10//CAPN7//RTN4//TNFSF14//STARD4//TRDN//MEST//ABHD5//TG//TNFAIP8L3//ACTR3//ACTR2//AVL9//RHOF//PEX5//DIXDC1//CNTN2//STON2//BEX4//PARD6B//ITGA5//NISCH//RRAS//SFRP1//CYP4F2//ATP6V1G2//TTK//SEPT7//BSN//CH25H//BRK1//CHRM1//TXNDC5//IL4R//ALAD//NRAS//PSMD7//TSPAN14//PAXIP1//WDR91//SPCS2//ITGA8//STATH//RBFOX1//FOXP3//MOGAT2//TNFSF4//SKP1//KRT2//POLR2M//GNAO1//SLC46A3//JPH1//PRKAA1//TULP4//TNKS2//PARVA//LRRC38//FERMT2//PALM//SKAP1//GRIN2B//PRKAR2B//FABP2//GRIPAP1//CLDN18//OXSR1//ZSWIM6//NUCB1//TP53INP2//UBE2D3//SHQ1//SESTD1//SLC35G1//COPS5//PAX8//GDF9// |
| GO:0002712 | regulation of B cell mediated immunity | Biological process | 4 | 46 | 368 | 17653 | 4.17131379962193 | 0.0152296835693089 | 0.772174740185072 | 1.81730912000806 | 0.0108695652173913 | TNFSF4//PAXIP1//FOXP3//NDFIP1// |
| GO:0002889 | regulation of immunoglobulin mediated immune response | Biological process | 4 | 46 | 368 | 17653 | 4.17131379962193 | 0.0152296835693089 | 0.772174740185072 | 1.81730912000806 | 0.0108695652173913 | TNFSF4//PAXIP1//FOXP3//NDFIP1// |
| GO:0016447 | somatic recombination of immunoglobulin gene segments | Biological process | 4 | 46 | 368 | 17653 | 4.17131379962193 | 0.0152296835693089 | 0.772174740185072 | 1.81730912000806 | 0.0108695652173913 | PAXIP1//FOXP3//NDFIP1//TNFSF4// |
| GO:0007411 | axon guidance | Biological process | 11 | 248 | 368 | 17653 | 2.12770643408135 | 0.0152846357215759 | 0.772174740185072 | 1.81574490734644 | 0.0298913043478261 | UNC5A//FZD3//SEMA4G//DLX5//ZSWIM6//EFNA3//NCAM1//NRAS//CNTN2//VLDLR//TNFRSF8// |
| GO:0097061 | dendritic spine organization | Biological process | 5 | 70 | 368 | 17653 | 3.42643633540373 | 0.015349727498091 | 0.772174740185072 | 1.81389933009727 | 0.0135869565217391 | CTNND2//LRRK2//ACTR2//GRIN2B//ZNF804A// |
| GO:0007275 | multicellular organism development | Biological process | 131 | 5351 | 368 | 17653 | 1.17437567541215 | 0.0158493757317981 | 0.772174740185072 | 1.79998783890835 | 0.355978260869565 | CLEC3A//DLX5//EVC//OTOR//EPAS1//HIF1A//ITGA5//NOS3//RTN4//VEZF1//JAM3//PROK1//ALDH1A2//PAXIP1//HIPK1//PAX8//ITGA8//SMAD1//SFRP1//SIM1//BRK1//TLX2//SEMA4G//FBXO45//MARK1//FZD3//AGTR1//SKIL//CDKN1B//NDP//LDOC1//PPP3CB//CCDC103//MICAL2//PARVA//OTP//CTNS//STON2//MLF1//FOXP3//RRAS//NDFIP1//FZD2//HEYL//UBE4B//KRT2//ACTL6A//CXXC4//PAX5//CHRM1//CHRNA3//FGF11//KIF2A//RBFOX1//SCN3B//PCDHB3//PURA//SMPD1//VLDLR//LRRC38//PARD6B//EFNA3//NCAM1//NRAS//CNTN2//UNC5A//TNFRSF8//BSN//NPTX1//FOXG1//GRIN2B//CA10//SLC5A3//BMP10//JPH1//PAXBP1//EMP2//LRRK2//MEA1//HOXC9//ZNF804A//BAG5//SEPT7//ABCC8//TMEM108//UCHL5//DIXDC1//COPS2//IFNK//RHOH//KMT2E//STATH//TG//GNAO1//NREP//PRDM12//KRTAP1-1//INSIG2//KCNQ4//PLLP//GAL3ST1//GPD2//MAEA//TNFSF4//CD46//SLC27A4//CD8A//KCNIP2//HMGB3//IL4R//VWC2//DDAH1//CLDN18//C2ORF49//PRKAA1//P2RX2//C11ORF88//PPP3CA//LRRTM1//PSMD7//PALM//CTNND2//CPEB3//ACTR2//BFSP1//ZSWIM6//CTH//GDF9//STRBP//ASF1B//TMEFF1// |
| GO:0007229 | integrin-mediated signaling pathway | Biological process | 6 | 97 | 368 | 17653 | 2.96722321828776 | 0.0159277312841396 | 0.772174740185072 | 1.79784607987667 | 0.016304347826087 | EMP2//FERMT2//ITGA5//TLN1//FERMT3//ITGA8// |
| GO:0097485 | neuron projection guidance | Biological process | 11 | 250 | 368 | 17653 | 2.1106847826087 | 0.0161318243263174 | 0.772174740185072 | 1.79231651605485 | 0.0298913043478261 | EFNA3//NCAM1//NRAS//CNTN2//VLDLR//UNC5A//TNFRSF8//FZD3//SEMA4G//DLX5//ZSWIM6// |
| GO:1901028 | regulation of mitochondrial outer membrane permeabilization involved in apoptotic signaling pathway | Biological process | 4 | 47 | 368 | 17653 | 4.08256244218316 | 0.0163799104665062 | 0.772174740185072 | 1.7856884764467 | 0.0108695652173913 | PMAIP1//YWHAB//YWHAZ//SLC25A5// |
| GO:1901615 | organic hydroxy compound metabolic process | Biological process | 18 | 497 | 368 | 17653 | 1.73734800104978 | 0.0169238776846822 | 0.772174740185072 | 1.77150012214124 | 0.0489130434782609 | SLC5A3//MOGAT2//HIF1A//IDI1//INSIG2//PRKAA1//CH25H//VLDLR//GPD2//CYP4F3//CYP4F2//TG//EPAS1//CTNS//ALDH1A2//STARD4//ACADL//PAX8// |
| GO:0051716 | cellular response to stimulus | Biological process | 178 | 7556 | 368 | 17653 | 1.13005285175041 | 0.0169649452500282 | 0.772174740185072 | 1.77044753758969 | 0.483695652173913 | LRRK2//GRIN2B//SMAD1//NCAM1//NRAS//MAPK4//PSMD7//YWHAB//PRKAA1//PROK1//CD81//COPS5//COPS2//PMAIP1//SKP1//CXCL11//UBE2D3//SKAP1//P2RX2//FERMT2//CTNND2//TRABD2B//MARK1//NDP//CXXC4//TNKS2//FZD2//GNAO1//PPP3CA//PPP3CB//FZD3//FAM13A//RALBP1//PRDM4//PTPRT//AKAP10//CHRM1//CHRNA3//ARHGAP36//EPAS1//FGF11//PDIA3//KCNIP3//KCNIP2//HIF1A//IGSF1//IL4R//MRC1//RASL11B//SMPD1//ABCC8//TG//TNFSF4//VLDLR//YWHAZ//NDFIP1//CAMLG//OGT//ACTL6A//TNFSF14//TNFRSF8//SH3BP5//CD8A//CARHSP1//PRKAR2B//POLR2M//OXSR1//UCHL5//PARG//POLR2E//PAXIP1//AOC3//FBXO45//RNF169//CDKN1B//CLEC1A//TSPAN14//TSPAN18//FAM83B//GDF9//SKIL//AGTR1//GPR160//GNAZ//GPR34//RIC8B//SFRP1//ARHGEF6//P2RY14//GPR3//PALM//HEYL//HIPK1//EVC//ITGA5//TLN1//FERMT3//ITGA8//DDAH1//NOS3//ARFIP2//RHOH//RHOF//RALGPS2//RRAS//EPS8L2//ATP6V1G2//PTBP1//CD46//UBE4B//PPP1R2//EBI3//BMP10//IFNK//TTK//PIP4K2C//MGRN1//NISCH//BRK1//NREP//LRRTM1//TNFAIP8L3//CPEB3//FOXP3//DLX5//HTRA4//VWC2//CTH//CREB3L3//JAM3//LARP1//RABL3//INSIG2//WDFY1//GPX8//HSPA13//NRBF2//TXNDC5//RNF149//CH25H//ACTR2//KDELR3//GOSR2//DNAJB12//UNC5A//ACTR3//PTPN18//PAX8//MCMDC2//NUDT15//EFNA3//ALDH1A2//RTN4//PAX5//CGNL1//TMEM108//DIXDC1//CNTN2//JPH1//TRDN//ALAD//VAMP3//ELK1//SEMA4G//PARVA//CLDN18//CASP7//BSN//LYPD6//NUP214//BAG5//BAG2//SLC25A5//LDOC1//FAM168A//UBXN2A//EIF4A2//EMP2// |
| GO:0051641 | cellular localization | Biological process | 73 | 2761 | 368 | 17653 | 1.26831507960254 | 0.0172062843133621 | 0.772174740185072 | 1.76431290517679 | 0.198369565217391 | PDIA3//SPRN//PPP3CA//NUP214//PLLP//PMAIP1//U2AF1//YWHAB//YWHAZ//GOLGA7B//KDELR3//GOSR2//TIMM23//TOMM34//AP1S1//SNX31//ARFIP2//RABL3//KCNIP3//TBC1D22B//MLPH//OTOR//SYS1//VAMP3//TLN1//CPLX1//TMEM108//MGRN1//P2RX2//TRDN//CHRNA3//SYT14//ACTR3//ACTR2//PEX5//HIF1A//TOMM40L//STARD4//VPS53//LRRK2//EMP2//TTK//SEPT7//MAPRE1//PPP3CB//IL4R//WDR91//SPCS2//STON2//CXCL11//SKP1//POLR2M//JPH1//PARD6B//CD81//TULP4//TNKS2//CNTN2//FERMT2//PALM//SCN3B//TSPAN14//SKAP1//CHRM1//GRIN2B//PRKAR2B//GRIPAP1//CLDN18//NUCB1//TP53INP2//PRKAA1//UBE2D3//RTN4// |
| GO:0000338 | protein deneddylation | Biological process | 2 | 10 | 368 | 17653 | 9.59402173913043 | 0.0174599936628631 | 0.772174740185072 | 1.7579559182584 | 0.00543478260869565 | COPS5//COPS2// |
| GO:0002638 | negative regulation of immunoglobulin production | Biological process | 2 | 10 | 368 | 17653 | 9.59402173913043 | 0.0174599936628631 | 0.772174740185072 | 1.7579559182584 | 0.00543478260869565 | FOXP3//NDFIP1// |
| GO:0002713 | negative regulation of B cell mediated immunity | Biological process | 2 | 10 | 368 | 17653 | 9.59402173913043 | 0.0174599936628631 | 0.772174740185072 | 1.7579559182584 | 0.00543478260869565 | FOXP3//NDFIP1// |
| GO:0002890 | negative regulation of immunoglobulin mediated immune response | Biological process | 2 | 10 | 368 | 17653 | 9.59402173913043 | 0.0174599936628631 | 0.772174740185072 | 1.7579559182584 | 0.00543478260869565 | FOXP3//NDFIP1// |
| GO:0006621 | protein retention in ER lumen | Biological process | 2 | 10 | 368 | 17653 | 9.59402173913043 | 0.0174599936628631 | 0.772174740185072 | 1.7579559182584 | 0.00543478260869565 | KDELR3//PDIA3// |
| GO:0021670 | lateral ventricle development | Biological process | 2 | 10 | 368 | 17653 | 9.59402173913043 | 0.0174599936628631 | 0.772174740185072 | 1.7579559182584 | 0.00543478260869565 | PAX5//UCHL5// |
| GO:0045625 | regulation of T-helper 1 cell differentiation | Biological process | 2 | 10 | 368 | 17653 | 9.59402173913043 | 0.0174599936628631 | 0.772174740185072 | 1.7579559182584 | 0.00543478260869565 | IL4R//TNFSF4// |
| GO:0060159 | regulation of dopamine receptor signaling pathway | Biological process | 2 | 10 | 368 | 17653 | 9.59402173913043 | 0.0174599936628631 | 0.772174740185072 | 1.7579559182584 | 0.00543478260869565 | LRRK2//PALM// |
| GO:1901569 | fatty acid derivative catabolic process | Biological process | 2 | 10 | 368 | 17653 | 9.59402173913043 | 0.0174599936628631 | 0.772174740185072 | 1.7579559182584 | 0.00543478260869565 | CYP4F3//CYP4F2// |
| GO:1902950 | regulation of dendritic spine maintenance | Biological process | 2 | 10 | 368 | 17653 | 9.59402173913043 | 0.0174599936628631 | 0.772174740185072 | 1.7579559182584 | 0.00543478260869565 | GRIN2B//ZNF804A// |
| GO:2001054 | negative regulation of mesenchymal cell apoptotic process | Biological process | 2 | 10 | 368 | 17653 | 9.59402173913043 | 0.0174599936628631 | 0.772174740185072 | 1.7579559182584 | 0.00543478260869565 | PAX8//HIF1A// |
| GO:0002381 | immunoglobulin production involved in immunoglobulin mediated immune response | Biological process | 4 | 48 | 368 | 17653 | 3.99750905797101 | 0.0175831598386073 | 0.772174740185072 | 1.75490307595413 | 0.0108695652173913 | PAXIP1//FOXP3//NDFIP1//TNFSF4// |
| GO:0060324 | face development | Biological process | 4 | 48 | 368 | 17653 | 3.99750905797101 | 0.0175831598386073 | 0.772174740185072 | 1.75490307595413 | 0.0108695652173913 | DLX5//RRAS//CRISPLD1//ALDH1A2// |
| GO:0106027 | neuron projection organization | Biological process | 5 | 73 | 368 | 17653 | 3.28562388326385 | 0.0181188803351823 | 0.781633034918104 | 1.74186864326385 | 0.0135869565217391 | CTNND2//LRRK2//ACTR2//GRIN2B//ZNF804A// |
| GO:0007628 | adult walking behavior | Biological process | 3 | 27 | 368 | 17653 | 5.33001207729469 | 0.0181452589800595 | 0.781633034918104 | 1.7412368289069 | 0.00815217391304348 | CTNS//SCN1A//CNTN2// |
| GO:0032673 | regulation of interleukin-4 production | Biological process | 3 | 27 | 368 | 17653 | 5.33001207729469 | 0.0181452589800595 | 0.781633034918104 | 1.7412368289069 | 0.00815217391304348 | FOXP3//NDFIP1//TNFSF4// |
| GO:0010646 | regulation of cell communication | Biological process | 90 | 3522 | 368 | 17653 | 1.2258119768906 | 0.0187616523709981 | 0.789561536245107 | 1.72672891521043 | 0.244565217391304 | LRRK2//PRKAA1//PROK1//CD81//AGTR1//SLC25A5//PPP3CB//ABCC8//RIC8B//CD46//PPP1R2//SKAP1//EBI3//FGF11//GDF9//BMP10//NDP//IFNK//CXCL11//TG//TNFSF4//TNFSF14//TRDN//HIPK1//TTK//CHRNA3//PIP4K2C//MGRN1//YWHAB//NREP//SMPD1//FOXP3//SFRP1//TRABD2B//CXXC4//ITGA8//HTRA4//SKIL//RASL11B//VWC2//HIF1A//ITGA5//NISCH//RALGPS2//WDFY1//OGT//EPS8L2//ARHGEF6//CTH//NDFIP1//RHOH//RNF149//PMAIP1//TSPAN14//EVC//COPS5//LRRTM1//PPP3CA//CPLX1//CPEB3//GRIN2B//CNTN2//P2RX2//PAX5//FAM13A//RALBP1//ARHGAP36//RHOF//CGNL1//TMEM108//RRAS//TNFAIP8L3//RTN4//PALM//JPH1//HEYL//CTNND2//PSMD7//DLX5//TNKS2//DIXDC1//LYPD6//YWHAZ//PTPN18//NOS3//BAG5//LDOC1//PAX8//EMP2//PDIA3// |
| GO:0007626 | locomotory behavior | Biological process | 9 | 191 | 368 | 17653 | 2.26037161393125 | 0.018990377267016 | 0.789561536245107 | 1.72146640738748 | 0.0244565217391304 | CTNS//SCN1A//CNTN2//PPP3CB//LRRK2//CHRNA3//GNAO1//LRRTM1//NOVA1// |
| GO:0043266 | regulation of potassium ion transport | Biological process | 6 | 101 | 368 | 17653 | 2.84970942746449 | 0.0190774597472146 | 0.789561536245107 | 1.719479454119 | 0.016304347826087 | NOS3//OXSR1//KCNIP3//KCNIP2//LRRC38//ABCC8// |
| GO:0032273 | positive regulation of protein polymerization | Biological process | 7 | 130 | 368 | 17653 | 2.58300585284281 | 0.019253832719346 | 0.789561536245107 | 1.7154828057306 | 0.0190217391304348 | RHOH//CDKN1B//MAPRE1//ACTR3//ACTR2//ARFIP2//BRK1// |
| GO:0072655 | establishment of protein localization to mitochondrion | Biological process | 9 | 192 | 368 | 17653 | 2.2485988451087 | 0.0195727854568638 | 0.789561536245107 | 1.70834736430301 | 0.0244565217391304 | PMAIP1//TIMM23//TOMM34//TOMM40L//YWHAB//YWHAZ//LRRK2//PRKAA1//UBE2D3// |
| GO:0050954 | sensory perception of mechanical stimulus | Biological process | 8 | 161 | 368 | 17653 | 2.38360788549824 | 0.0197984667544067 | 0.789561536245107 | 1.70336844134875 | 0.0217391304347826 | CDKN1B//P2RX2//NDP//USP53//OTOR//EPS8L2//KCNQ4//SCN1A// |
| GO:0099173 | postsynapse organization | Biological process | 6 | 102 | 368 | 17653 | 2.82177109974425 | 0.019926686887387 | 0.789561536245107 | 1.70056490331335 | 0.016304347826087 | CTNND2//LRRK2//ACTR2//TMEM108//GRIN2B//ZNF804A// |
| GO:0090092 | regulation of transmembrane receptor protein serine/threonine kinase signaling pathway | Biological process | 10 | 225 | 368 | 17653 | 2.13200483091787 | 0.0199573079559956 | 0.789561536245107 | 1.6998980411401 | 0.0271739130434783 | GDF9//BMP10//TTK//NREP//ITGA8//HTRA4//SKIL//RASL11B//VWC2//SFRP1// |
| GO:0021799 | cerebral cortex radially oriented cell migration | Biological process | 3 | 28 | 368 | 17653 | 5.13965450310559 | 0.0200154292983299 | 0.789561536245107 | 1.69863509055032 | 0.00815217391304348 | RTN4//FBXO45//DIXDC1// |
| GO:0046636 | negative regulation of alpha-beta T cell activation | Biological process | 3 | 28 | 368 | 17653 | 5.13965450310559 | 0.0200154292983299 | 0.789561536245107 | 1.69863509055032 | 0.00815217391304348 | IL4R//TNFSF4//FOXP3// |
| GO:0071634 | regulation of transforming growth factor beta production | Biological process | 3 | 28 | 368 | 17653 | 5.13965450310559 | 0.0200154292983299 | 0.789561536245107 | 1.69863509055032 | 0.00815217391304348 | HIF1A//FOXP3//CD46// |
| GO:0090659 | walking behavior | Biological process | 3 | 28 | 368 | 17653 | 5.13965450310559 | 0.0200154292983299 | 0.789561536245107 | 1.69863509055032 | 0.00815217391304348 | CTNS//SCN1A//CNTN2// |
| GO:0046635 | positive regulation of alpha-beta T cell activation | Biological process | 4 | 50 | 368 | 17653 | 3.83760869565217 | 0.0201513070764607 | 0.789561536245107 | 1.69569677891821 | 0.0108695652173913 | FOXP3//TNFSF4//IL4R//EBI3// |
| GO:0055078 | sodium ion homeostasis | Biological process | 4 | 50 | 368 | 17653 | 3.83760869565217 | 0.0201513070764607 | 0.789561536245107 | 1.69569677891821 | 0.0108695652173913 | AGTR1//ATP1B4//SCN7A//CYP4F2// |
| GO:2001238 | positive regulation of extrinsic apoptotic signaling pathway | Biological process | 4 | 50 | 368 | 17653 | 3.83760869565217 | 0.0201513070764607 | 0.789561536245107 | 1.69569677891821 | 0.0108695652173913 | PMAIP1//SFRP1//SKIL//PDIA3// |
| GO:0030509 | BMP signaling pathway | Biological process | 8 | 162 | 368 | 17653 | 2.36889425657542 | 0.0204635134369804 | 0.789561536245107 | 1.68901979900629 | 0.0217391304347826 | VWC2//SFRP1//SKIL//DLX5//GDF9//BMP10//SMAD1//UBE2D3// |
| GO:0010959 | regulation of metal ion transport | Biological process | 14 | 363 | 368 | 17653 | 1.85008683674692 | 0.0205757261027188 | 0.789561536245107 | 1.68664482990233 | 0.0380434782608696 | NOS3//P2RX2//SCN3B//TRDN//CXCL11//BEST1//GNAO1//JPH1//OXSR1//KCNIP3//KCNIP2//LRRC38//ABCC8//SESTD1// |
| GO:0071310 | cellular response to organic substance | Biological process | 70 | 2656 | 368 | 17653 | 1.264272443033 | 0.0209855307994168 | 0.789561536245107 | 1.6780800414469 | 0.190217391304348 | FERMT2//GDF9//SMAD1//SKIL//CHRM1//ATP6V1G2//POLR2E//PTBP1//HIPK1//NREP//HIF1A//LRRTM1//IL4R//IFNK//YWHAZ//CPEB3//DLX5//BMP10//UBE2D3//ITGA8//HTRA4//RASL11B//VWC2//SFRP1//ITGA5//CTH//CREB3L3//NOS3//PSMD7//TNFSF4//TNFSF14//TNFRSF8//HSPA13//PPP3CB//ACTR2//KDELR3//TLN1//GOSR2//AGTR1//OGT//TMEM108//NCAM1//HEYL//CXCL11//EBI3//SKP1//DNAJB12//MRC1//ALDH1A2//PPP3CA//PTPN18//ACTR3//VAMP3//ALAD//PRKAA1//PAX8//PRKAR2B//ELK1//CDKN1B//CLDN18//CASP7//LARP1//CHRNA3//LRRK2//COPS5//UBXN2A//EIF4A2//SLC25A5//MAPK4//ABCC8// |
| GO:0010872 | regulation of cholesterol esterification | Biological process | 2 | 11 | 368 | 17653 | 8.72183794466403 | 0.0210490750092902 | 0.789561536245107 | 1.67676698425528 | 0.00543478260869565 | STARD4//AGTR1// |
| GO:0043619 | regulation of transcription from RNA polymerase II promoter in response to oxidative stress | Biological process | 2 | 11 | 368 | 17653 | 8.72183794466403 | 0.0210490750092902 | 0.789561536245107 | 1.67676698425528 | 0.00543478260869565 | EPAS1//HIF1A// |
| GO:0045628 | regulation of T-helper 2 cell differentiation | Biological process | 2 | 11 | 368 | 17653 | 8.72183794466403 | 0.0210490750092902 | 0.789561536245107 | 1.67676698425528 | 0.00543478260869565 | IL4R//TNFSF4// |
| GO:0070585 | protein localization to mitochondrion | Biological process | 9 | 195 | 368 | 17653 | 2.21400501672241 | 0.0213975503403123 | 0.789561536245107 | 1.66963594321999 | 0.0244565217391304 | PMAIP1//TIMM23//TOMM34//TOMM40L//YWHAB//YWHAZ//LRRK2//PRKAA1//UBE2D3// |
| GO:1902905 | positive regulation of supramolecular fiber organization | Biological process | 9 | 195 | 368 | 17653 | 2.21400501672241 | 0.0213975503403123 | 0.789561536245107 | 1.66963594321999 | 0.0244565217391304 | RHOH//CDKN1B//MAPRE1//ACTR3//ACTR2//ARFIP2//SFRP1//BMP10//BRK1// |
| GO:0045010 | actin nucleation | Biological process | 4 | 51 | 368 | 17653 | 3.76236146632566 | 0.0215173755440311 | 0.789561536245107 | 1.6672107003036 | 0.0108695652173913 | ACTR3//ACTR2//ARFIP2//BRK1// |
| GO:0045589 | regulation of regulatory T cell differentiation | Biological process | 3 | 29 | 368 | 17653 | 4.96242503748126 | 0.0219877498906129 | 0.789561536245107 | 1.6578192117529 | 0.00815217391304348 | FOXP3//TNFSF4//CD46// |
| GO:0070306 | lens fiber cell differentiation | Biological process | 3 | 29 | 368 | 17653 | 4.96242503748126 | 0.0219877498906129 | 0.789561536245107 | 1.6578192117529 | 0.00815217391304348 | BFSP1//CDKN1B//SKIL// |
| GO:0071604 | transforming growth factor beta production | Biological process | 3 | 29 | 368 | 17653 | 4.96242503748126 | 0.0219877498906129 | 0.789561536245107 | 1.6578192117529 | 0.00815217391304348 | HIF1A//FOXP3//CD46// |
| GO:2000516 | positive regulation of CD4-positive, alpha-beta T cell activation | Biological process | 3 | 29 | 368 | 17653 | 4.96242503748126 | 0.0219877498906129 | 0.789561536245107 | 1.6578192117529 | 0.00815217391304348 | FOXP3//TNFSF4//IL4R// |
| GO:0060284 | regulation of cell development | Biological process | 27 | 858 | 368 | 17653 | 1.50954887503801 | 0.022151352485463 | 0.789561536245107 | 1.6545997521027 | 0.0733695652173913 | OTP//HIF1A//FZD3//SFRP1//ZNF804A//LRRK2//TNFRSF8//BAG5//RTN4//CNTN2//EFNA3//NREP//DIXDC1//HEYL//VWC2//CHRNA3//SEMA4G//SKIL//TLX2//PPP3CA//ABCC8//BMP10//CPEB3//ACTR2//VLDLR//ZSWIM6//CLDN18// |
| GO:0048813 | dendrite morphogenesis | Biological process | 7 | 134 | 368 | 17653 | 2.50590120051914 | 0.0223177362931758 | 0.789561536245107 | 1.65134985835631 | 0.0190217391304348 | CHRNA3//TLX2//PPP3CA//CTNND2//LRRK2//ACTR2//VLDLR// |
| GO:0034613 | cellular protein localization | Biological process | 49 | 1762 | 368 | 17653 | 1.3340155085624 | 0.0224937144976617 | 0.789561536245107 | 1.64793882145928 | 0.133152173913043 | PDIA3//SPRN//PPP3CA//NUP214//PMAIP1//U2AF1//YWHAB//YWHAZ//GOLGA7B//KDELR3//GOSR2//TIMM23//TOMM34//AP1S1//SNX31//ARFIP2//RABL3//KCNIP3//TBC1D22B//MLPH//TLN1//PEX5//TOMM40L//LRRK2//EMP2//TTK//MAPRE1//SYS1//VAMP3//SPCS2//SKP1//CD81//TULP4//TNKS2//CNTN2//FERMT2//PALM//SCN3B//TSPAN14//SKAP1//CHRM1//GRIPAP1//CLDN18//NUCB1//TP53INP2//PRKAA1//UBE2D3//RTN4//SEPT7// |
| GO:0035967 | cellular response to topologically incorrect protein | Biological process | 8 | 166 | 368 | 17653 | 2.31181246726035 | 0.0232827514856117 | 0.789561536245107 | 1.63296569737235 | 0.0217391304347826 | CTH//CREB3L3//HSPA13//KDELR3//TLN1//GOSR2//DNAJB12//COPS5// |
| GO:0044265 | cellular macromolecule catabolic process | Biological process | 33 | 1103 | 368 | 17653 | 1.43518910875478 | 0.0235093765223965 | 0.789561536245107 | 1.62875888837711 | 0.0896739130434783 | PNRC1//EIF4A2//UBE4B//RNF217//FBXO45//RLIM//UCHL5//TP53INP2//UBE2D3//UBE2E1//NUDT15//PMAIP1//PSMD7//BUB3//FBXL3//SKP1//OGT//BAG5//LRRK2//VLDLR//DNAJB12//MAEA//UBXN2B//UBXN2A//CARHSP1//YWHAB//YWHAZ//NUP214//LARP1//CPEB3//ALAD//WDR91//CD81// |
| GO:0048522 | positive regulation of cellular process | Biological process | 125 | 5142 | 368 | 17653 | 1.16613449765782 | 0.0235346827881558 | 0.789561536245107 | 1.62829165094914 | 0.339673913043478 | LRRK2//PRKAA1//PROK1//CD81//PAXIP1//VAMP3//HIF1A//NRAS//OTP//FZD3//CXCL11//PMAIP1//CDKN1B//CHRNA3//HEYL//CHRM1//EMP2//HIPK1//GDF9//SLC25A5//PGGT1B//BRK1//PURA//SFRP1//TTK//TNFSF4//ALDH1A2//CASP7//SKAP1//TP53INP2//P2RX2//CAPN7//RTN4//AGTR1//MICAL2//TNFSF14//BMP10//STARD4//ABHD5//CNTN2//ZNF804A//CPEB3//LARP1//RBM3//DLX5//ELK1//FZD2//NDP//FOXP3//PPP3CB//KMT2E//PAX8//CREB5//EIF4G2//MAPRE1//ITGA5//SEMA4G//FERMT3//ITGA8//RHOH//PAXBP1//NOS3//NDFIP1//OXSR1//NEK7//TNKS2//RNF217//TRABD2B//INSIG2//IFNK//WDFY1//PRKAR2B//ACTR3//ACTR2//ARFIP2//SMPD1//CD46//TNFRSF8//IL4R//RRP1B//SHQ1//ARHGEF6//CTH//TNFAIP8L3//EBI3//DDAH1//VEZF1//VWC2//SMAD1//TSPAN14//PPP3CA//VLDLR//OGT//EVC//COPS5//PRDM4//EPAS1//ATP1B4//TLX2//MCIDAS//PAX5//SIM1//CREB3L3//SKIL//TMEM108//PALM//PRDM12//LRRTM1//GRIN2B//TRDN//PSMD7//DIXDC1//EPS8L2//LSM11//YWHAB//YWHAZ//CALCOCO2//SEPT7//EFNA3//LRRC38//ABCC8//UBE2D3//FAM168A//PIP4K2C//PDIA3// |
| GO:0008104 | protein localization | Biological process | 69 | 2628 | 368 | 17653 | 1.25948915525114 | 0.0236285534973935 | 0.789561536245107 | 1.62656286437392 | 0.1875 | PDIA3//SPRN//PPP3CA//NUP214//PMAIP1//U2AF1//YWHAB//YWHAZ//GOLGA7B//KDELR3//GOSR2//TIMM23//TOMM34//AP1S1//SNX31//ARFIP2//RABL3//KCNIP3//TBC1D22B//MLPH//TLN1//SLC25A5//PPP3CB//ABCC8//OTOR//VPS53//SCAMP1//PEX5//CPLX1//TOMM40L//LRRK2//ATP6V1G2//EMP2//VLDLR//TTK//SEPT7//MAPRE1//LRRTM1//BSN//HIF1A//SYS1//VAMP3//SPCS2//ITGA8//SFRP1//FOXP3//TNFSF4//NDFIP1//SKP1//CD81//TULP4//TNKS2//CNTN2//FERMT2//PALM//SCN3B//TSPAN14//SKAP1//IL4R//CHRM1//GRIPAP1//CLDN18//NUCB1//TP53INP2//PRKAA1//UBE2D3//RTN4//AKAP10//PLLP// |
| GO:0007612 | learning | Biological process | 7 | 136 | 368 | 17653 | 2.46904971227621 | 0.023969571360448 | 0.789561536245107 | 1.62033973223487 | 0.0190217391304348 | ACTR2//CTNS//HIF1A//ABCC8//PPP3CB//PRKAR2B//CNTN2// |
| GO:0050773 | regulation of dendrite development | Biological process | 7 | 136 | 368 | 17653 | 2.46904971227621 | 0.023969571360448 | 0.789561536245107 | 1.62033973223487 | 0.0190217391304348 | CHRNA3//TLX2//PPP3CA//CPEB3//LRRK2//ACTR2//VLDLR// |
| GO:0031345 | negative regulation of cell projection organization | Biological process | 8 | 167 | 368 | 17653 | 2.29796927883364 | 0.0240281965236017 | 0.789561536245107 | 1.61927882467013 | 0.0217391304347826 | LRRK2//TNFRSF8//BAG5//RTN4//SEMA4G//TLX2//PPP3CA//GRIN2B// |
| GO:0002828 | regulation of type 2 immune response | Biological process | 3 | 30 | 368 | 17653 | 4.79701086956522 | 0.0240622950225966 | 0.789561536245107 | 1.61866295271872 | 0.00815217391304348 | NDFIP1//TNFSF4//IL4R// |
| GO:0045622 | regulation of T-helper cell differentiation | Biological process | 3 | 30 | 368 | 17653 | 4.79701086956522 | 0.0240622950225966 | 0.789561536245107 | 1.61866295271872 | 0.00815217391304348 | IL4R//TNFSF4//FOXP3// |
| GO:0060325 | face morphogenesis | Biological process | 3 | 30 | 368 | 17653 | 4.79701086956522 | 0.0240622950225966 | 0.789561536245107 | 1.61866295271872 | 0.00815217391304348 | DLX5//RRAS//CRISPLD1// |
| GO:1900739 | regulation of protein insertion into mitochondrial membrane involved in apoptotic signaling pathway | Biological process | 3 | 30 | 368 | 17653 | 4.79701086956522 | 0.0240622950225966 | 0.789561536245107 | 1.61866295271872 | 0.00815217391304348 | PMAIP1//YWHAB//YWHAZ// |
| GO:1900740 | positive regulation of protein insertion into mitochondrial membrane involved in apoptotic signaling pathway | Biological process | 3 | 30 | 368 | 17653 | 4.79701086956522 | 0.0240622950225966 | 0.789561536245107 | 1.61866295271872 | 0.00815217391304348 | PMAIP1//YWHAB//YWHAZ// |
| GO:0043550 | regulation of lipid kinase activity | Biological process | 4 | 53 | 368 | 17653 | 3.62038556193601 | 0.0244160712832946 | 0.789561536245107 | 1.6123242157904 | 0.0108695652173913 | CD81//WDR91//TNFAIP8L3//NRBF2// |
| GO:0070727 | cellular macromolecule localization | Biological process | 49 | 1772 | 368 | 17653 | 1.32648720433801 | 0.024622454406092 | 0.789561536245107 | 1.60866865807029 | 0.133152173913043 | PDIA3//SPRN//PPP3CA//NUP214//PMAIP1//U2AF1//YWHAB//YWHAZ//GOLGA7B//KDELR3//GOSR2//TIMM23//TOMM34//AP1S1//SNX31//ARFIP2//RABL3//KCNIP3//TBC1D22B//MLPH//TLN1//PEX5//TOMM40L//LRRK2//EMP2//TTK//SEPT7//MAPRE1//SYS1//VAMP3//SPCS2//SKP1//CD81//TULP4//TNKS2//CNTN2//FERMT2//PALM//SCN3B//TSPAN14//SKAP1//CHRM1//GRIPAP1//CLDN18//NUCB1//TP53INP2//PRKAA1//UBE2D3//RTN4// |
| GO:0006527 | arginine catabolic process | Biological process | 2 | 12 | 368 | 17653 | 7.99501811594203 | 0.0249151597091519 | 0.789561536245107 | 1.60353632460446 | 0.00543478260869565 | DDAH1//NOS3// |
| GO:0032700 | negative regulation of interleukin-17 production | Biological process | 2 | 12 | 368 | 17653 | 7.99501811594203 | 0.0249151597091519 | 0.789561536245107 | 1.60353632460446 | 0.00543478260869565 | FOXP3//TNFSF4// |
| GO:0033631 | cell-cell adhesion mediated by integrin | Biological process | 2 | 12 | 368 | 17653 | 7.99501811594203 | 0.0249151597091519 | 0.789561536245107 | 1.60353632460446 | 0.00543478260869565 | FERMT3//ITGA5// |
| GO:0045161 | neuronal ion channel clustering | Biological process | 2 | 12 | 368 | 17653 | 7.99501811594203 | 0.0249151597091519 | 0.789561536245107 | 1.60353632460446 | 0.00543478260869565 | KCNIP2//CNTN2// |
| GO:0045591 | positive regulation of regulatory T cell differentiation | Biological process | 2 | 12 | 368 | 17653 | 7.99501811594203 | 0.0249151597091519 | 0.789561536245107 | 1.60353632460446 | 0.00543478260869565 | FOXP3//CD46// |
| GO:0060766 | negative regulation of androgen receptor signaling pathway | Biological process | 2 | 12 | 368 | 17653 | 7.99501811594203 | 0.0249151597091519 | 0.789561536245107 | 1.60353632460446 | 0.00543478260869565 | HEYL//SFRP1// |
| GO:1900363 | regulation of mRNA polyadenylation | Biological process | 2 | 12 | 368 | 17653 | 7.99501811594203 | 0.0249151597091519 | 0.789561536245107 | 1.60353632460446 | 0.00543478260869565 | CPEB3//SNRPA// |
| GO:2001053 | regulation of mesenchymal cell apoptotic process | Biological process | 2 | 12 | 368 | 17653 | 7.99501811594203 | 0.0249151597091519 | 0.789561536245107 | 1.60353632460446 | 0.00543478260869565 | PAX8//HIF1A// |
| GO:0030001 | metal ion transport | Biological process | 27 | 868 | 368 | 17653 | 1.49215775896614 | 0.0252003360613089 | 0.789561536245107 | 1.59859366760739 | 0.0733695652173913 | NOS3//CDKN1B//KCNIP2//ABCC8//KCNQ4//SCN3B//SCN1A//SCN7A//SLC5A3//SLC23A2//PPP3CA//SLC39A2//ATP1B4//P2RX2//TRDN//ATP6V1G2//CXCL11//BEST1//GNAO1//JPH1//LRRC38//KCNIP3//GRIN2B//OXSR1//SESTD1//SLC35G1//NDFIP1// |
| GO:0072659 | protein localization to plasma membrane | Biological process | 10 | 234 | 368 | 17653 | 2.05000464511334 | 0.02533895679317 | 0.789561536245107 | 1.59621126902226 | 0.0271739130434783 | SYS1//VAMP3//GRIPAP1//EMP2//KCNIP3//GOLGA7B//PALM//SCN3B//TSPAN14//SKAP1// |
| GO:0006140 | regulation of nucleotide metabolic process | Biological process | 9 | 201 | 368 | 17653 | 2.14791531473069 | 0.0254087608643167 | 0.789561536245107 | 1.59501651412758 | 0.0244565217391304 | NUP214//OGT//PALM//CXCL11//FZD2//NOS3//HIF1A//PRKAA1//ME2// |
| GO:0032502 | developmental process | Biological process | 149 | 6267 | 368 | 17653 | 1.1405052171138 | 0.0254223930776159 | 0.789561536245107 | 1.5947835705607 | 0.404891304347826 | ERVFRD-1//RHOH//CLEC3A//DLX5//EVC//OTOR//EPAS1//HIF1A//ITGA5//NOS3//RTN4//VEZF1//JAM3//PROK1//GDF9//ALDH1A2//PAXIP1//TP53INP2//SFRP1//HIPK1//PAX8//ITGA8//SMAD1//SIM1//BRK1//TLX2//FZD3//SEMA4G//FBXO45//MARK1//AGTR1//SKIL//CDKN1B//NDP//LDOC1//PPP3CB//CCDC103//MICAL2//PARVA//OTP//CTNS//STON2//MLF1//FOXP3//RRAS//NDFIP1//FZD2//HEYL//UBE4B//KRT2//ACTL6A//GRIN2B//HMGB3//PAX5//STRBP//ASF1B//TMEFF1//TNFRSF8//FOXG1//CXXC4//CHRM1//CHRNA3//FGF11//KIF2A//RBFOX1//SCN3B//PCDHB3//PURA//SMPD1//VLDLR//LRRC38//PARD6B//EFNA3//NCAM1//NRAS//CNTN2//UNC5A//BSN//NPTX1//CA10//SLC5A3//MEST//BMP10//JPH1//PAXBP1//EMP2//GNAO1//LRRK2//FERMT2//PALM//RHOF//EVPLL//MEA1//HOXC9//ZNF804A//BAG5//SEPT7//ABCC8//TMEM108//UCHL5//DIXDC1//COPS2//ELK1//ABHD5//IFNK//KMT2E//STATH//TG//NREP//PRDM12//KRTAP1-1//FERMT3//VAMP3//POLR2E//GPD2//TNKS2//INSIG2//KCNQ4//PLLP//GAL3ST1//MAEA//TNFSF4//CD46//SLC27A4//CD8A//KCNIP2//IL4R//TNFSF14//VWC2//DDAH1//BFSP1//MCMDC2//CLDN18//C2ORF49//PRKAA1//P2RX2//C11ORF88//PPP3CA//PTBP1//LRRTM1//PSMD7//CRISPLD1//CTNND2//CPEB3//ACTR2//ZNF322//ZSWIM6//MCIDAS//CTH// |
| GO:0031323 | regulation of cellular metabolic process | Biological process | 153 | 6454 | 368 | 17653 | 1.1371903672815 | 0.025442330563787 | 0.789561536245107 | 1.59444310903646 | 0.415760869565217 | CDKN1B//CPEB3//HEYL//KCNIP3//PAX5//FOXP3//RLIM//SKIL//UBE2D3//VLDLR//BHLHE40//COPS2//PAXBP1//LRRK2//PRKAA1//PROK1//CD81//RBFOX1//PTBP1//PAXIP1//VAMP3//LRRTM1//PPP1R3F//NUP214//OGT//MCIDAS//PARG//KRBOX1//ZBTB6//PNRC1//ZNF618//CTNND2//HIPK1//EWSR1//ZBTB43//ATP1B4//MYCBP//NRBF2//HIF1A//HMGB3//IGSF1//RHOH//MLF1//UCHL5//PFDN1//ASF1B//ZFP64//IFNK//ZNF124//ZNF322//ELK1//EPAS1//FOXG1//OTP//CARHSP1//HOXC9//PURA//CREBZF//PRDM12//SIM1//VEZF1//ACTL6A//CREB5//RBM3//EIF4A2//EIF4G2//CEP85//PSMD7//PMAIP1//CHRNA3//PALM//NUDT15//CASP7//DRAM1//PIP4K2C//TP53INP2//DLAT//AGTR1//CTNS//MICAL2//GDF9//BMP10//TTK//STARD4//ABHD5//RRP1B//ZCCHC9//PPP1R26//A2ML1//CNTN2//LARP1//ATP6V1G2//DLX5//FZD2//NDP//PPP3CB//KMT2E//SFRP1//PAX8//SKAP1//TNFSF4//YWHAB//MEX3B//SMAD1//VWC2//CXCL11//NOS3//UBXN2A//BAG5//NDFIP1//EMP2//OXSR1//NEK7//TNKS2//RNF217//PPP1R2//INSIG2//PRKAR2B//SMPD1//TNFRSF8//TNFSF14//RNF149//YWHAZ//WDR91//TNFAIP8L3//UBXN2B//EBI3//DDAH1//ACADL//MAEA//COPS5//PRDM4//TLX2//PPP3CA//CREB3L3//ITGA5//CTH//NOVA1//POLR2E//SH3BP5//RRAS//SNRPA//CALCOCO2//ALAD//ME2//EFNA3//BEX4//TRABD2B//FAM168A//SHQ1//ITGA8//RNF169//GRIN2B// |
| GO:0048583 | regulation of response to stimulus | Biological process | 105 | 4243 | 368 | 17653 | 1.18709908391315 | 0.0254881096471269 | 0.789561536245107 | 1.59366237325042 | 0.285326086956522 | LRRK2//PRKAA1//PROK1//CD81//PPP3CB//NRAS//PSMD7//SKP1//CREB3L3//FOXP3//CXCL11//TNFSF4//UBE2D3//SKAP1//NDFIP1//PARG//CD46//RIC8B//PPP1R2//EBI3//FGF11//GDF9//BMP10//NDP//IFNK//TG//TNFSF14//NOS3//HIPK1//TTK//CHRNA3//PIP4K2C//PPP3CA//MGRN1//YWHAB//NREP//SMPD1//SFRP1//TRABD2B//CXXC4//ITGA8//HTRA4//SKIL//RASL11B//VWC2//HIF1A//ITGA5//RALGPS2//WDFY1//OGT//EPS8L2//ARHGEF6//ACTR3//ACTR2//BRK1//CTH//RHOH//IL4R//RNF149//PMAIP1//RNF135//TSPAN14//PAXIP1//EVC//COPS5//LRRTM1//RTN4//SEMA4G//AGTR1//CD8A//P2RX2//PAX5//FAM13A//RALBP1//ARHGAP36//RHOF//CGNL1//TMEM108//RRAS//TNFAIP8L3//PALM//CNTN2//JPH1//TRDN//HEYL//CTNND2//ABCC8//JAM3//DLX5//TNKS2//DIXDC1//LYPD6//NUP214//BAG5//BAG2//DDAH1//YWHAZ//SLC25A5//PTPN18//ZSWIM6//LDOC1//FAM168A//RNF169//EMP2//PDIA3// |
| GO:0019218 | regulation of steroid metabolic process | Biological process | 6 | 108 | 368 | 17653 | 2.66500603864734 | 0.0255649304178587 | 0.789561536245107 | 1.59235538499029 | 0.016304347826087 | STARD4//AGTR1//INSIG2//IDI1//PRKAA1//ACADL// |
| GO:0060079 | excitatory postsynaptic potential | Biological process | 6 | 108 | 368 | 17653 | 2.66500603864734 | 0.0255649304178587 | 0.789561536245107 | 1.59235538499029 | 0.016304347826087 | LRRK2//TMEM108//CHRNA3//P2RX2//GRIN2B//PPP3CA// |
| GO:0010977 | negative regulation of neuron projection development | Biological process | 7 | 138 | 368 | 17653 | 2.43326638311279 | 0.0257036537216799 | 0.789561536245107 | 1.59000513821038 | 0.0190217391304348 | RTN4//SEMA4G//TLX2//PPP3CA//LRRK2//TNFRSF8//BAG5// |
| GO:0007405 | neuroblast proliferation | Biological process | 4 | 54 | 368 | 17653 | 3.55334138486312 | 0.0259495845955851 | 0.789561536245107 | 1.58586959000393 | 0.0108695652173913 | OTP//HIF1A//FZD3//LRRK2// |
| GO:0008284 | positive regulation of cell proliferation | Biological process | 28 | 911 | 368 | 17653 | 1.47438314322531 | 0.0262263516864069 | 0.789561536245107 | 1.58126211934718 | 0.0760869565217391 | HIF1A//NRAS//OTP//FZD3//CD81//FOXP3//RTN4//CD46//TNFSF4//EBI3//DLX5//SFRP1//BMP10//AGTR1//PAXBP1//CDKN1B//CHRM1//EMP2//HIPK1//GDF9//SLC25A5//PGGT1B//PRKAA1//BRK1//PURA//TTK//PROK1//ALDH1A2// |
| GO:0034694 | response to prostaglandin | Biological process | 3 | 31 | 368 | 17653 | 4.64226858345021 | 0.0262389857851416 | 0.789561536245107 | 1.58105295574566 | 0.00815217391304348 | PRKAA1//SFRP1//TNFSF4// |
| GO:0045066 | regulatory T cell differentiation | Biological process | 3 | 31 | 368 | 17653 | 4.64226858345021 | 0.0262389857851416 | 0.789561536245107 | 1.58105295574566 | 0.00815217391304348 | FOXP3//TNFSF4//CD46// |
| GO:0071353 | cellular response to interleukin-4 | Biological process | 3 | 31 | 368 | 17653 | 4.64226858345021 | 0.0262389857851416 | 0.789561536245107 | 1.58105295574566 | 0.00815217391304348 | IL4R//ALAD//MRC1// |
| GO:0045665 | negative regulation of neuron differentiation | Biological process | 9 | 203 | 368 | 17653 | 2.12675358749197 | 0.0268570194242551 | 0.789561536245107 | 1.57094218673152 | 0.0244565217391304 | LRRK2//TNFRSF8//BAG5//RTN4//SEMA4G//TLX2//PPP3CA//CNTN2//DIXDC1// |
| GO:0070887 | cellular response to chemical stimulus | Biological process | 81 | 3175 | 368 | 17653 | 1.22380434782609 | 0.0269686263069276 | 0.789561536245107 | 1.56914117451541 | 0.220108695652174 | CXCL11//P2RX2//AOC3//FERMT2//GDF9//SMAD1//SKIL//CHRM1//ATP6V1G2//POLR2E//PTBP1//HIPK1//TNFSF14//NREP//HIF1A//LRRTM1//IL4R//IFNK//YWHAZ//NOS3//CPEB3//DLX5//BMP10//UBE2D3//ITGA8//HTRA4//RASL11B//VWC2//SFRP1//ITGA5//CTH//CREB3L3//PSMD7//TNFSF4//TNFRSF8//LRRK2//GPX8//HSPA13//RNF149//PPP3CA//PPP3CB//CH25H//ACTR2//KDELR3//TLN1//GOSR2//AGTR1//EPAS1//OGT//TMEM108//NCAM1//HEYL//EBI3//PRKAA1//SKP1//DNAJB12//MRC1//CDKN1B//ALAD//ALDH1A2//MAPK4//ABCC8//PTPN18//ACTR3//VAMP3//PAX8//PRKAR2B//ELK1//PMAIP1//PARVA//CLDN18//CASP7//LARP1//JAM3//CHRNA3//DDAH1//BAG5//COPS5//UBXN2A//EIF4A2//SLC25A5// |
| GO:0070542 | response to fatty acid | Biological process | 5 | 81 | 368 | 17653 | 2.96111782071927 | 0.0270545392916654 | 0.789561536245107 | 1.56775985721434 | 0.0135869565217391 | PRKAA1//SFRP1//TNFSF4//ALAD//INSIG2// |
| GO:1901379 | regulation of potassium ion transmembrane transport | Biological process | 5 | 81 | 368 | 17653 | 2.96111782071927 | 0.0270545392916654 | 0.789561536245107 | 1.56775985721434 | 0.0135869565217391 | OXSR1//LRRC38//ABCC8//KCNIP3//KCNIP2// |
| GO:0009894 | regulation of catabolic process | Biological process | 28 | 914 | 368 | 17653 | 1.46954381124536 | 0.0272093953924002 | 0.789561536245107 | 1.56528110837319 | 0.0760869565217391 | NUP214//OGT//UCHL5//NUDT15//LRRK2//DRAM1//PIP4K2C//EIF4G2//PRKAA1//TP53INP2//ABHD5//LARP1//HIF1A//ATP6V1G2//BAG5//RNF217//UBXN2A//CARHSP1//PSMD7//YWHAB//YWHAZ//CDKN1B//NDFIP1//CPEB3//CALCOCO2//ALAD//WDR91//CD81// |
| GO:0010975 | regulation of neuron projection development | Biological process | 16 | 450 | 368 | 17653 | 1.7056038647343 | 0.0272529371327182 | 0.789561536245107 | 1.56458668561512 | 0.0434782608695652 | ZNF804A//LRRK2//TNFRSF8//BAG5//RTN4//CNTN2//CHRNA3//SEMA4G//SKIL//TLX2//PPP3CA//CPEB3//ACTR2//VLDLR//ZSWIM6//SFRP1// |
| GO:0021537 | telencephalon development | Biological process | 10 | 237 | 368 | 17653 | 2.02405521922583 | 0.0273448663283909 | 0.789561536245107 | 1.56312419527285 | 0.0271739130434783 | TMEM108//PAX5//UCHL5//LRRK2//FBXO45//DIXDC1//RTN4//CNTN2//DLX5//HIF1A// |
| GO:0070646 | protein modification by small protein removal | Biological process | 12 | 306 | 368 | 17653 | 1.88118073316283 | 0.0274201608289352 | 0.789561536245107 | 1.56193000224839 | 0.0326086956521739 | COPS5//COPS2//USP54//HIF1A//SMAD1//UCHL5//USP53//PSMD7//TNKS2//RNF135//OGT//ACTL6A// |
| GO:0030163 | protein catabolic process | Biological process | 27 | 875 | 368 | 17653 | 1.48022049689441 | 0.0275219443355804 | 0.789561536245107 | 1.56032088786439 | 0.0733695652173913 | UBE4B//RNF217//FBXO45//RLIM//UCHL5//TP53INP2//UBE2D3//UBE2E1//NUDT15//PMAIP1//PSMD7//BUB3//FBXL3//SKP1//OGT//BAG5//LRRK2//VLDLR//DNAJB12//UBXN2A//MAEA//UBXN2B//CDKN1B//NDFIP1//ALAD//WDR91//CD81// |
| GO:0060688 | regulation of morphogenesis of a branching structure | Biological process | 4 | 55 | 368 | 17653 | 3.48873517786561 | 0.0275397211838 | 0.789561536245107 | 1.56004046091824 | 0.0108695652173913 | SFRP1//PAX8//LRRK2//RTN4// |
| GO:0097345 | mitochondrial outer membrane permeabilization | Biological process | 4 | 55 | 368 | 17653 | 3.48873517786561 | 0.0275397211838 | 0.789561536245107 | 1.56004046091824 | 0.0108695652173913 | PMAIP1//YWHAB//YWHAZ//SLC25A5// |
| GO:0007267 | cell-cell signaling | Biological process | 46 | 1658 | 368 | 17653 | 1.33089565741858 | 0.0276550214699063 | 0.789561536245107 | 1.55822600003126 | 0.125 | AGTR1//FERMT2//CTNND2//TRABD2B//MARK1//NDP//PRKAA1//SKP1//CXXC4//TNKS2//FZD2//GNAO1//PPP3CA//PPP3CB//FZD3//CPLX1//CHRNA3//GRIN2B//KCNIP2//NOVA1//NPTX1//PCDHB3//BSN//CHRM1//P2RX2//SLC25A5//ABCC8//SYT14//FOXP3//SFRP1//NISCH//HIF1A//CPEB3//CNTN2//LRRK2//DIXDC1//PSMD7//LRRTM1//SCN3B//DLX5//TMEM108//PAX8//GDF9//EFNA3//FGF11//CXCL11// |
| GO:0051603 | proteolysis involved in cellular protein catabolic process | Biological process | 22 | 679 | 368 | 17653 | 1.55425978100788 | 0.0278086853390348 | 0.789561536245107 | 1.55581954200597 | 0.0597826086956522 | UBE4B//RNF217//FBXO45//RLIM//UCHL5//TP53INP2//UBE2D3//UBE2E1//NUDT15//PMAIP1//PSMD7//BUB3//FBXL3//SKP1//OGT//BAG5//LRRK2//DNAJB12//MAEA//UBXN2B//UBXN2A//ALAD// |
| GO:0048872 | homeostasis of number of cells | Biological process | 10 | 238 | 368 | 17653 | 2.0155507855316 | 0.0280380148571965 | 0.789561536245107 | 1.55225273846162 | 0.0271739130434783 | JAM3//FOXP3//HIF1A//SKIL//EPAS1//KMT2E//PMAIP1//PPP3CB//TNFSF14//MAEA// |
| GO:0042147 | retrograde transport, endosome to Golgi | Biological process | 5 | 82 | 368 | 17653 | 2.92500662778367 | 0.028337167962316 | 0.789561536245107 | 1.54764355563398 | 0.0135869565217391 | LRRK2//AP1S1//VPS53//VAMP3//GOSR2// |
| GO:0001844 | protein insertion into mitochondrial membrane involved in apoptotic signaling pathway | Biological process | 3 | 32 | 368 | 17653 | 4.49719769021739 | 0.0285175985833413 | 0.789561536245107 | 1.54488704845249 | 0.00815217391304348 | PMAIP1//YWHAB//YWHAZ// |
| GO:0006732 | coenzyme metabolic process | Biological process | 14 | 380 | 368 | 17653 | 1.76731979405034 | 0.0289227949110309 | 0.789561536245107 | 1.5387597419519 | 0.0380434782608696 | DLAT//RPE//NUP214//OGT//GPD2//DBI//NFS1//RFK//SLC23A2//AADAT//CYP4F2//HIF1A//PRKAA1//ME2// |
| GO:0032352 | positive regulation of hormone metabolic process | Biological process | 2 | 13 | 368 | 17653 | 7.38001672240803 | 0.0290452046884684 | 0.789561536245107 | 1.53692555860042 | 0.00543478260869565 | HIF1A//PAX8// |
| GO:0035437 | maintenance of protein localization in endoplasmic reticulum | Biological process | 2 | 13 | 368 | 17653 | 7.38001672240803 | 0.0290452046884684 | 0.789561536245107 | 1.53692555860042 | 0.00543478260869565 | KDELR3//PDIA3// |
| GO:0046541 | saliva secretion | Biological process | 2 | 13 | 368 | 17653 | 7.38001672240803 | 0.0290452046884684 | 0.789561536245107 | 1.53692555860042 | 0.00543478260869565 | CHRM1//STATH// |
| GO:0090179 | planar cell polarity pathway involved in neural tube closure | Biological process | 2 | 13 | 368 | 17653 | 7.38001672240803 | 0.0290452046884684 | 0.789561536245107 | 1.53692555860042 | 0.00543478260869565 | FZD2//SFRP1// |
| GO:0097152 | mesenchymal cell apoptotic process | Biological process | 2 | 13 | 368 | 17653 | 7.38001672240803 | 0.0290452046884684 | 0.789561536245107 | 1.53692555860042 | 0.00543478260869565 | PAX8//HIF1A// |
| GO:0032879 | regulation of localization | Biological process | 70 | 2700 | 368 | 17653 | 1.24366948470209 | 0.0290760575058503 | 0.789561536245107 | 1.53646448087799 | 0.190217391304348 | AGTR1//NOS3//LRRTM1//CXCL11//SLC25A5//PPP3CB//ABCC8//P2RX2//EMP2//BMP10//CAPN7//HIF1A//RTN4//SCN3B//TNFSF14//TRDN//MEST//ABHD5//CHRNA3//CPLX1//SYT14//STON2//KIF2A//RHOF//BEX4//PARD6B//MAPRE1//ITGA5//SEMA4G//FERMT3//NISCH//RRAS//SFRP1//TBC1D22B//CYP4F2//NDFIP1//LRRK2//SCN1A//SCN7A//KCNQ4//CHRM1//IL4R//PPP3CA//NUP214//FOXP3//TNFSF4//CDKN1B//BEST1//GNAO1//JPH1//PRKAA1//JAM3//CLDN18//PMAIP1//YWHAB//YWHAZ//OXSR1//KCNIP3//KCNIP2//ZSWIM6//VAMP3//NUCB1//LRRC38//TP53INP2//UBE2D3//SHQ1//SESTD1//CD81//PAX8//GDF9// |
| GO:0002562 | somatic diversification of immune receptors via germline recombination within a single locus | Biological process | 4 | 56 | 368 | 17653 | 3.42643633540373 | 0.0291868082302642 | 0.789561536245107 | 1.53481339537767 | 0.0108695652173913 | PAXIP1//FOXP3//NDFIP1//TNFSF4// |
| GO:0016444 | somatic cell DNA recombination | Biological process | 4 | 56 | 368 | 17653 | 3.42643633540373 | 0.0291868082302642 | 0.789561536245107 | 1.53481339537767 | 0.0108695652173913 | PAXIP1//FOXP3//NDFIP1//TNFSF4// |
| GO:0016445 | somatic diversification of immunoglobulins | Biological process | 4 | 56 | 368 | 17653 | 3.42643633540373 | 0.0291868082302642 | 0.789561536245107 | 1.53481339537767 | 0.0108695652173913 | PAXIP1//FOXP3//NDFIP1//TNFSF4// |
| GO:0048814 | regulation of dendrite morphogenesis | Biological process | 5 | 83 | 368 | 17653 | 2.88976558407543 | 0.0296577157191046 | 0.799104109196432 | 1.52786230201942 | 0.0135869565217391 | TLX2//PPP3CA//LRRK2//ACTR2//CHRNA3// |
| GO:0065009 | regulation of molecular function | Biological process | 85 | 3373 | 368 | 17653 | 1.20885242784774 | 0.0307635878722338 | 0.815896105042191 | 1.51196301535157 | 0.230978260869565 | CDKN1B//LRRK2//PRKAA1//PROK1//CD81//LRRTM1//CEP85//PMAIP1//CHRNA3//PALM//CASP7//EBI3//FGF11//GDF9//BMP10//NDP//IFNK//CXCL11//TG//TNFSF4//TNFSF14//RRP1B//ZCCHC9//PPP1R26//A2ML1//UCHL5//RHOH//NOS3//FOXP3//EMP2//OXSR1//NDFIP1//AGTR1//PPP1R2//RALBP1//FAM13A//ARHGAP36//GNAO1//RIC8B//SFRP1//ARHGEF6//RFK//PRKAR2B//PPP3CA//YWHAB//SMPD1//RLIM//BHLHE40//NRBF2//WDR91//TNFAIP8L3//UBXN2B//UBXN2A//VLDLR//BAG2//DDAH1//HIF1A//ABHD5//CREBZF//COPS5//FZD2//CTH//BAG5//NEK7//JPH1//TRDN//SH3BP5//TBC1D22B//LYPD6//EIF4A2//AOC3//EFNA3//MAPRE1//LDOC1//LRRC38//ABCC8//PPP1R3F//SCN3B//HEYL//GRIN2B//KCNIP2//KCNIP3//NCAM1//RALGPS2//EPS8L2// |
| GO:0097306 | cellular response to alcohol | Biological process | 4 | 57 | 368 | 17653 | 3.36632341723875 | 0.0308911275342795 | 0.815896105042191 | 1.51016623954643 | 0.0108695652173913 | PRKAA1//SFRP1//TNFSF4//LARP1// |
| GO:0007274 | neuromuscular synaptic transmission | Biological process | 3 | 33 | 368 | 17653 | 4.36091897233202 | 0.0308977732958312 | 0.815896105042191 | 1.51007281766832 | 0.00815217391304348 | CHRNA3//CHRM1//P2RX2// |
| GO:0019433 | triglyceride catabolic process | Biological process | 3 | 33 | 368 | 17653 | 4.36091897233202 | 0.0308977732958312 | 0.815896105042191 | 1.51007281766832 | 0.00815217391304348 | ABHD5//PNPLA5//FABP2// |
| GO:0032633 | interleukin-4 production | Biological process | 3 | 33 | 368 | 17653 | 4.36091897233202 | 0.0308977732958312 | 0.815896105042191 | 1.51007281766832 | 0.00815217391304348 | FOXP3//NDFIP1//TNFSF4// |
| GO:0042552 | myelination | Biological process | 6 | 113 | 368 | 17653 | 2.54708541746826 | 0.03100477583851 | 0.815896105042191 | 1.50857140421221 | 0.016304347826087 | CNTN2//TG//TNFRSF8//PLLP//JAM3//GAL3ST1// |
| GO:0003007 | heart morphogenesis | Biological process | 10 | 243 | 368 | 17653 | 1.97407854714618 | 0.0316923072064449 | 0.825051141486548 | 1.49904614293171 | 0.0271739130434783 | HIF1A//CCDC103//MICAL2//PARVA//FZD2//HEYL//UBE4B//BMP10//RTN4//ALDH1A2// |
| GO:0019222 | regulation of metabolic process | Biological process | 162 | 6920 | 368 | 17653 | 1.12299965443579 | 0.031962581896729 | 0.825051141486548 | 1.49535814618495 | 0.440217391304348 | CDKN1B//CPEB3//HEYL//KCNIP3//PAX5//FOXP3//RLIM//SKIL//UBE2D3//VLDLR//BHLHE40//COPS2//PAXBP1//PNRC1//LRRK2//PRKAA1//PROK1//CD81//EIF4A2//RBFOX1//PTBP1//PAXIP1//VAMP3//LRRTM1//PPP1R3F//NUP214//OGT//MCIDAS//PARG//KRBOX1//ZBTB6//ZNF618//CTNND2//HIPK1//EWSR1//ZBTB43//ATP1B4//MYCBP//NRBF2//HIF1A//HMGB3//IGSF1//RHOH//MLF1//UCHL5//PFDN1//ASF1B//ZFP64//IFNK//ZNF124//ZNF322//ELK1//EPAS1//FOXG1//OTP//CARHSP1//HOXC9//PURA//CREBZF//PRDM12//SIM1//VEZF1//ACTL6A//CREB5//RBM3//EIF4G2//CEP85//PSMD7//PMAIP1//CHRNA3//PALM//NUDT15//CASP7//PYURF//UBXN2A//MAPK4//DRAM1//PIP4K2C//TP53INP2//DLAT//SMAD1//CD46//ALDH1A2//ZNF804A//SFRP1//NDFIP1//AGTR1//CTNS//MICAL2//GDF9//BMP10//TTK//STARD4//INSIG2//ABHD5//RRP1B//ZCCHC9//PPP1R26//A2ML1//CNTN2//LARP1//ATP6V1G2//DLX5//FZD2//NDP//PPP3CB//KMT2E//PAX8//SKAP1//TNFSF4//YWHAB//MEX3B//TNFAIP8L3//VWC2//CXCL11//NOS3//BAG5//EMP2//OXSR1//NEK7//TNKS2//RNF217//PPP1R2//RFK//PRKAR2B//SMPD1//TNFRSF8//TNFSF14//RNF149//YWHAZ//WDR91//UBXN2B//EBI3//DDAH1//IDI1//ACADL//MAEA//POLR2E//COPS5//PRDM4//TLX2//PPP3CA//CREB3L3//ITGA5//CTH//NOVA1//PGGT1B//SH3BP5//RRAS//SNRPA//CALCOCO2//ALAD//ME2//AOC3//EFNA3//BEX4//TRABD2B//FAM168A//SHQ1//ITGA8//RNF169//GRIN2B// |
| GO:0051051 | negative regulation of transport | Biological process | 16 | 460 | 368 | 17653 | 1.66852551984877 | 0.032404157793865 | 0.825051141486548 | 1.48939926167358 | 0.0434782608695652 | LRRTM1//NOS3//CYP4F2//NDFIP1//PPP3CA//SFRP1//ABCC8//FOXP3//GNAO1//TRDN//OXSR1//SLC25A5//LRRK2//VAMP3//SESTD1//PRKAA1// |
| GO:0007605 | sensory perception of sound | Biological process | 7 | 145 | 368 | 17653 | 2.31579835082459 | 0.0324438205148584 | 0.825051141486548 | 1.48886800986089 | 0.0190217391304348 | CDKN1B//P2RX2//NDP//USP53//OTOR//EPS8L2//KCNQ4// |
| GO:0051235 | maintenance of location | Biological process | 12 | 314 | 368 | 17653 | 1.83325256161728 | 0.0325122343856691 | 0.825051141486548 | 1.48795318282558 | 0.0326086956521739 | KDELR3//PDIA3//TLN1//STARD4//TRDN//MEST//ABHD5//YWHAB//CXCL11//SKP1//POLR2M//JPH1// |
| GO:0099175 | regulation of postsynapse organization | Biological process | 4 | 58 | 368 | 17653 | 3.30828335832084 | 0.032652916118456 | 0.825051141486548 | 1.48607802732609 | 0.0108695652173913 | LRRK2//ACTR2//GRIN2B//ZNF804A// |
| GO:0007272 | ensheathment of neurons | Biological process | 6 | 115 | 368 | 17653 | 2.50278827977316 | 0.0333768612422762 | 0.825051141486548 | 1.4765545067596 | 0.016304347826087 | CNTN2//TG//TNFRSF8//PLLP//JAM3//GAL3ST1// |
| GO:0008366 | axon ensheathment | Biological process | 6 | 115 | 368 | 17653 | 2.50278827977316 | 0.0333768612422762 | 0.825051141486548 | 1.4765545067596 | 0.016304347826087 | CNTN2//TG//TNFRSF8//PLLP//JAM3//GAL3ST1// |
| GO:0070670 | response to interleukin-4 | Biological process | 3 | 34 | 368 | 17653 | 4.23265664961637 | 0.0333790211152307 | 0.825051141486548 | 1.47652640374087 | 0.00815217391304348 | IL4R//ALAD//MRC1// |
| GO:0010893 | positive regulation of steroid biosynthetic process | Biological process | 2 | 14 | 368 | 17653 | 6.85287267080745 | 0.0334265877225069 | 0.825051141486548 | 1.47590795511318 | 0.00543478260869565 | PRKAA1//STARD4// |
| GO:0034433 | steroid esterification | Biological process | 2 | 14 | 368 | 17653 | 6.85287267080745 | 0.0334265877225069 | 0.825051141486548 | 1.47590795511318 | 0.00543478260869565 | STARD4//AGTR1// |
| GO:0034434 | sterol esterification | Biological process | 2 | 14 | 368 | 17653 | 6.85287267080745 | 0.0334265877225069 | 0.825051141486548 | 1.47590795511318 | 0.00543478260869565 | STARD4//AGTR1// |
| GO:0034435 | cholesterol esterification | Biological process | 2 | 14 | 368 | 17653 | 6.85287267080745 | 0.0334265877225069 | 0.825051141486548 | 1.47590795511318 | 0.00543478260869565 | STARD4//AGTR1// |
| GO:0035589 | G-protein coupled purinergic nucleotide receptor signaling pathway | Biological process | 2 | 14 | 368 | 17653 | 6.85287267080745 | 0.0334265877225069 | 0.825051141486548 | 1.47590795511318 | 0.00543478260869565 | GPR34//P2RY14// |
| GO:0045064 | T-helper 2 cell differentiation | Biological process | 2 | 14 | 368 | 17653 | 6.85287267080745 | 0.0334265877225069 | 0.825051141486548 | 1.47590795511318 | 0.00543478260869565 | IL4R//TNFSF4// |
| GO:0046851 | negative regulation of bone remodeling | Biological process | 2 | 14 | 368 | 17653 | 6.85287267080745 | 0.0334265877225069 | 0.825051141486548 | 1.47590795511318 | 0.00543478260869565 | CLDN18//SFRP1// |
| GO:0090178 | regulation of establishment of planar polarity involved in neural tube closure | Biological process | 2 | 14 | 368 | 17653 | 6.85287267080745 | 0.0334265877225069 | 0.825051141486548 | 1.47590795511318 | 0.00543478260869565 | FZD2//SFRP1// |
| GO:0034446 | substrate adhesion-dependent cell spreading | Biological process | 5 | 86 | 368 | 17653 | 2.78895980788675 | 0.0338495783188441 | 0.829437312211387 | 1.47044673716908 | 0.0135869565217391 | FERMT2//PARVA//FERMT3//ITGA8//VAMP3// |
| GO:1901655 | cellular response to ketone | Biological process | 5 | 86 | 368 | 17653 | 2.78895980788675 | 0.0338495783188441 | 0.829437312211387 | 1.47044673716908 | 0.0135869565217391 | PRKAA1//SFRP1//TNFSF4//ELK1//LARP1// |
| GO:1902041 | regulation of extrinsic apoptotic signaling pathway via death domain receptors | Biological process | 4 | 59 | 368 | 17653 | 3.25221075902727 | 0.0344723668792082 | 0.841648437559874 | 1.46252889682733 | 0.0108695652173913 | NOS3//PMAIP1//SFRP1//SKIL// |
| GO:0031329 | regulation of cellular catabolic process | Biological process | 25 | 814 | 368 | 17653 | 1.47328343659865 | 0.0347503769862092 | 0.845384171070981 | 1.45904047964531 | 0.0679347826086956 | NUP214//OGT//UCHL5//NUDT15//LRRK2//DRAM1//PIP4K2C//EIF4G2//PRKAA1//TP53INP2//ABHD5//LARP1//HIF1A//ATP6V1G2//BAG5//RNF217//CARHSP1//PSMD7//YWHAB//YWHAZ//CPEB3//CALCOCO2//ALAD//WDR91//CD81// |
| GO:0006479 | protein methylation | Biological process | 8 | 180 | 368 | 17653 | 2.13200483091787 | 0.035286539910766 | 0.847159595533499 | 1.45239092512923 | 0.0217391304347826 | PAXIP1//ZFP64//PAXBP1//KMT2E//PRDM4//PAX5//PRDM12//OGT// |
| GO:0008213 | protein alkylation | Biological process | 8 | 180 | 368 | 17653 | 2.13200483091787 | 0.035286539910766 | 0.847159595533499 | 1.45239092512923 | 0.0217391304347826 | PAXIP1//ZFP64//PAXBP1//KMT2E//PRDM4//PAX5//PRDM12//OGT// |
| GO:0045981 | positive regulation of nucleotide metabolic process | Biological process | 5 | 87 | 368 | 17653 | 2.7569027986007 | 0.0353244131214619 | 0.847159595533499 | 1.45192504477248 | 0.0135869565217391 | CXCL11//FZD2//NOS3//HIF1A//PRKAA1// |
| GO:1900544 | positive regulation of purine nucleotide metabolic process | Biological process | 5 | 87 | 368 | 17653 | 2.7569027986007 | 0.0353244131214619 | 0.847159595533499 | 1.45192504477248 | 0.0135869565217391 | CXCL11//FZD2//NOS3//HIF1A//PRKAA1// |
| GO:0048568 | embryonic organ development | Biological process | 15 | 428 | 368 | 17653 | 1.68119539821211 | 0.0354866572494735 | 0.847395230170446 | 1.44993490813285 | 0.0407608695652174 | EPAS1//HIF1A//CCDC103//MICAL2//ALDH1A2//DLX5//INSIG2//PAX8//FZD3//ITGA8//KCNQ4//HIPK1//PAX5//HOXC9//FZD2// |
| GO:0002707 | negative regulation of lymphocyte mediated immunity | Biological process | 3 | 35 | 368 | 17653 | 4.11172360248447 | 0.0359607320802777 | 0.847395230170446 | 1.44417147558206 | 0.00815217391304348 | PPP3CB//FOXP3//NDFIP1// |
| GO:0048741 | skeletal muscle fiber development | Biological process | 3 | 35 | 368 | 17653 | 4.11172360248447 | 0.0359607320802777 | 0.847395230170446 | 1.44417147558206 | 0.00815217391304348 | P2RX2//C11ORF88//PPP3CA// |
| GO:0051204 | protein insertion into mitochondrial membrane | Biological process | 3 | 35 | 368 | 17653 | 4.11172360248447 | 0.0359607320802777 | 0.847395230170446 | 1.44417147558206 | 0.00815217391304348 | PMAIP1//YWHAB//YWHAZ// |
| GO:2000648 | positive regulation of stem cell proliferation | Biological process | 3 | 35 | 368 | 17653 | 4.11172360248447 | 0.0359607320802777 | 0.847395230170446 | 1.44417147558206 | 0.00815217391304348 | OTP//HIF1A//FZD3// |
| GO:0071242 | cellular response to ammonium ion | Biological process | 4 | 60 | 368 | 17653 | 3.19800724637681 | 0.0363496292777712 | 0.853585218074884 | 1.43950001406049 | 0.0108695652173913 | CHRM1//CASP7//CHRNA3//LRRK2// |
| GO:0034620 | cellular response to unfolded protein | Biological process | 7 | 149 | 368 | 17653 | 2.25362926758097 | 0.0367812457621318 | 0.857857381948834 | 1.43437356551509 | 0.0190217391304348 | CTH//CREB3L3//KDELR3//TLN1//GOSR2//COPS5//HSPA13// |
| GO:1904018 | positive regulation of vasculature development | Biological process | 9 | 215 | 368 | 17653 | 2.00805106167846 | 0.036785249262925 | 0.857857381948834 | 1.43432629676037 | 0.0244565217391304 | HIPK1//DDAH1//HIF1A//NOS3//RRAS//AGTR1//ITGA5//SMAD1//CTH// |
| GO:0043269 | regulation of ion transport | Biological process | 20 | 622 | 368 | 17653 | 1.54244722494058 | 0.0372839011258089 | 0.858421169902707 | 1.42847865241322 | 0.0543478260869565 | NOS3//P2RX2//SCN3B//TRDN//CHRNA3//CYP4F2//SCN1A//SCN7A//KCNQ4//CHRM1//CXCL11//BEST1//GNAO1//JPH1//OXSR1//KCNIP3//KCNIP2//LRRC38//ABCC8//SESTD1// |
| GO:0007179 | transforming growth factor beta receptor signaling pathway | Biological process | 8 | 182 | 368 | 17653 | 2.10857620640229 | 0.0372887589372063 | 0.858421169902707 | 1.42842207080046 | 0.0217391304347826 | NREP//ITGA8//HTRA4//SKIL//RASL11B//FERMT2//GDF9//SMAD1// |
| GO:0048839 | inner ear development | Biological process | 8 | 182 | 368 | 17653 | 2.10857620640229 | 0.0372887589372063 | 0.858421169902707 | 1.42842207080046 | 0.0217391304347826 | DLX5//INSIG2//PAX8//FZD3//ITGA8//KCNQ4//FZD2//CDKN1B// |
| GO:0060322 | head development | Biological process | 23 | 741 | 368 | 17653 | 1.48895074224022 | 0.0377715436051599 | 0.858421169902707 | 1.4228352665491 | 0.0625 | CTNS//FOXG1//GRIN2B//CA10//ITGA8//TMEM108//PAX5//UCHL5//LRRK2//FBXO45//DIXDC1//RTN4//CNTN2//OTP//DLX5//ALDH1A2//HIF1A//GNAO1//SMAD1//FZD3//RRAS//CRISPLD1//SFRP1// |
| GO:0002830 | positive regulation of type 2 immune response | Biological process | 2 | 15 | 368 | 17653 | 6.39601449275362 | 0.0380470956357292 | 0.858421169902707 | 1.41967848994514 | 0.00543478260869565 | IL4R//TNFSF4// |
| GO:0050774 | negative regulation of dendrite morphogenesis | Biological process | 2 | 15 | 368 | 17653 | 6.39601449275362 | 0.0380470956357292 | 0.858421169902707 | 1.41967848994514 | 0.00543478260869565 | TLX2//PPP3CA// |
| GO:0090177 | establishment of planar polarity involved in neural tube closure | Biological process | 2 | 15 | 368 | 17653 | 6.39601449275362 | 0.0380470956357292 | 0.858421169902707 | 1.41967848994514 | 0.00543478260869565 | FZD2//SFRP1// |
| GO:1903818 | positive regulation of voltage-gated potassium channel activity | Biological process | 2 | 15 | 368 | 17653 | 6.39601449275362 | 0.0380470956357292 | 0.858421169902707 | 1.41967848994514 | 0.00543478260869565 | LRRC38//ABCC8// |
| GO:0002292 | T cell differentiation involved in immune response | Biological process | 4 | 61 | 368 | 17653 | 3.14558089807555 | 0.0382848100682676 | 0.858421169902707 | 1.41697350310858 | 0.0108695652173913 | CD46//TNFSF4//IL4R//FOXP3// |
| GO:1902110 | positive regulation of mitochondrial membrane permeability involved in apoptotic process | Biological process | 4 | 61 | 368 | 17653 | 3.14558089807555 | 0.0382848100682676 | 0.858421169902707 | 1.41697350310858 | 0.0108695652173913 | PMAIP1//YWHAB//YWHAZ//SLC25A5// |
| GO:0046718 | viral entry into host cell | Biological process | 6 | 119 | 368 | 17653 | 2.41866094263792 | 0.0384663670372208 | 0.858421169902707 | 1.41491882877487 | 0.016304347826087 | TMPRSS2//ITGA5//CD46//MRC1//NCAM1//CD81// |
| GO:0035418 | protein localization to synapse | Biological process | 3 | 36 | 368 | 17653 | 3.99750905797101 | 0.0386421823098957 | 0.858421169902707 | 1.41293835486569 | 0.00815217391304348 | GRIPAP1//LRRTM1//BSN// |
| GO:0060323 | head morphogenesis | Biological process | 3 | 36 | 368 | 17653 | 3.99750905797101 | 0.0386421823098957 | 0.858421169902707 | 1.41293835486569 | 0.00815217391304348 | DLX5//RRAS//CRISPLD1// |
| GO:0051495 | positive regulation of cytoskeleton organization | Biological process | 9 | 217 | 368 | 17653 | 1.98954367862152 | 0.0386550784579629 | 0.858421169902707 | 1.41279344090782 | 0.0244565217391304 | RHOH//CDKN1B//MAPRE1//ACTR3//ACTR2//ARFIP2//SFRP1//BMP10//BRK1// |
| GO:1901342 | regulation of vasculature development | Biological process | 14 | 396 | 368 | 17653 | 1.69591293368467 | 0.0388363443675498 | 0.858421169902707 | 1.41076165661469 | 0.0380434782608696 | EFNA3//ABCC8//EMP2//SFRP1//PROK1//HIPK1//DDAH1//HIF1A//NOS3//RRAS//AGTR1//ITGA5//SMAD1//CTH// |
| GO:0048598 | embryonic morphogenesis | Biological process | 19 | 586 | 368 | 17653 | 1.55534482119009 | 0.0388402895150419 | 0.858421169902707 | 1.41071754152622 | 0.0516304347826087 | TLX2//SMAD1//FZD3//HIF1A//CCDC103//MICAL2//ALDH1A2//DLX5//ITGA5//INSIG2//PAX8//ITGA8//KCNQ4//HIPK1//PAX5//HOXC9//FZD2//SFRP1//C2ORF49// |
| GO:0002822 | regulation of adaptive immune response based on somatic recombination of immune receptors built from immunoglobulin superfamily domains | Biological process | 6 | 120 | 368 | 17653 | 2.39850543478261 | 0.0398117247658934 | 0.868948251592414 | 1.39998900704077 | 0.016304347826087 | PPP3CB//FOXP3//TNFSF4//IL4R//PAXIP1//NDFIP1// |
| GO:0031124 | mRNA 3'-end processing | Biological process | 5 | 90 | 368 | 17653 | 2.66500603864734 | 0.0399844229300852 | 0.868948251592414 | 1.39810916749937 | 0.0135869565217391 | PAPOLB//LSM11//SNRPA//CPEB3//U2AF1// |
| GO:0044089 | positive regulation of cellular component biogenesis | Biological process | 17 | 511 | 368 | 17653 | 1.5958744575853 | 0.0400590268230029 | 0.868948251592414 | 1.39729960548012 | 0.046195652173913 | BRK1//RHOH//CDKN1B//MAPRE1//TRABD2B//PMAIP1//ACTR3//ACTR2//ARFIP2//PALM//SFRP1//LRRTM1//PAXIP1//BMP10//EPS8L2//SEPT7//PIP4K2C// |
| GO:0016239 | positive regulation of macroautophagy | Biological process | 4 | 62 | 368 | 17653 | 3.09484572230014 | 0.0402779740595204 | 0.868948251592414 | 1.39493238213278 | 0.0108695652173913 | CALCOCO2//PIP4K2C//LARP1//HIF1A// |
| GO:0072091 | regulation of stem cell proliferation | Biological process | 4 | 62 | 368 | 17653 | 3.09484572230014 | 0.0402779740595204 | 0.868948251592414 | 1.39493238213278 | 0.0108695652173913 | OTP//HIF1A//FZD3//LRRK2// |
| GO:1901565 | organonitrogen compound catabolic process | Biological process | 35 | 1238 | 368 | 17653 | 1.35618239446513 | 0.0409369588467545 | 0.868948251592414 | 1.38788442364544 | 0.0951086956521739 | CTH//NUDT15//DPYS//UBE4B//RNF217//FBXO45//RLIM//UCHL5//TP53INP2//UBE2D3//UBE2E1//DDAH1//NOS3//AADAT//SMPD1//PMAIP1//PSMD7//BUB3//FBXL3//SKP1//OGT//BAG5//LRRK2//VLDLR//DNAJB12//UBXN2A//ACADL//MAEA//UBXN2B//CDKN1B//NDFIP1//UPB1//ALAD//WDR91//CD81// |
| GO:0009893 | positive regulation of metabolic process | Biological process | 83 | 3329 | 368 | 17653 | 1.196010520198 | 0.0410192060382776 | 0.868948251592414 | 1.38701275002645 | 0.22554347826087 | LRRK2//PRKAA1//PROK1//CD81//PAXIP1//VAMP3//PMAIP1//CHRNA3//HEYL//CASP7//TP53INP2//HIF1A//SMAD1//CD46//ALDH1A2//ZNF804A//AGTR1//MICAL2//GDF9//BMP10//TTK//STARD4//ABHD5//CNTN2//CPEB3//LARP1//RBM3//DLX5//ELK1//FZD2//NDP//FOXP3//PPP3CB//KMT2E//SFRP1//PAX8//SKAP1//CREB5//CXCL11//PAXBP1//NOS3//NDFIP1//EMP2//OXSR1//NEK7//TNKS2//RNF217//INSIG2//IFNK//RFK//PRKAR2B//SMPD1//TNFRSF8//TNFAIP8L3//EBI3//DDAH1//CDKN1B//POLR2E//VLDLR//OGT//COPS5//PRDM4//EPAS1//RRP1B//ATP1B4//TLX2//MCIDAS//PAX5//PPP3CA//SIM1//VEZF1//CREB3L3//ITGA5//PRDM12//CALCOCO2//EFNA3//TRABD2B//FAM168A//TNFSF4//ITGA8//PIP4K2C//GRIN2B//PYURF// |
| GO:0003231 | cardiac ventricle development | Biological process | 6 | 121 | 368 | 17653 | 2.37868307581746 | 0.0411865912943946 | 0.868948251592414 | 1.38524414985697 | 0.016304347826087 | FZD2//HEYL//HIF1A//UBE4B//PAX8//BMP10// |
| GO:0042092 | type 2 immune response | Biological process | 3 | 37 | 368 | 17653 | 3.88946827262045 | 0.0414225409491266 | 0.868948251592414 | 1.38276326405344 | 0.00815217391304348 | NDFIP1//TNFSF4//IL4R// |
| GO:0043370 | regulation of CD4-positive, alpha-beta T cell differentiation | Biological process | 3 | 37 | 368 | 17653 | 3.88946827262045 | 0.0414225409491266 | 0.868948251592414 | 1.38276326405344 | 0.00815217391304348 | FOXP3//TNFSF4//IL4R// |
| GO:0045581 | negative regulation of T cell differentiation | Biological process | 3 | 37 | 368 | 17653 | 3.88946827262045 | 0.0414225409491266 | 0.868948251592414 | 1.38276326405344 | 0.00815217391304348 | TNFSF4//IL4R//FOXP3// |
| GO:0009628 | response to abiotic stimulus | Biological process | 34 | 1198 | 368 | 17653 | 1.3614221165711 | 0.0416134089386863 | 0.868948251592414 | 1.3807667056774 | 0.0923913043478261 | CDKN1B//EPAS1//ALAD//P2RX2//HIF1A//PRKAA1//STRBP//CTNS//ABCC8//NOS3//UBE4B//ELK1//PAXIP1//USP53//ANO3//TNFSF14//TNFRSF8//HSPA13//FBXL3//BHLHE40//BEST1//PRDM12//SCN1A//FZD3//PSMD7//UBE2D3//PALM//PMAIP1//SFRP1//OXSR1//NUP214//BAG5//BAG2//DDAH1// |
| GO:0042391 | regulation of membrane potential | Biological process | 15 | 438 | 368 | 17653 | 1.64281194163192 | 0.0420115063029442 | 0.868948251592414 | 1.37663174675696 | 0.0407608695652174 | USP53//PMAIP1//CTNS//SCN1A//SCN7A//SCN3B//LRRK2//CHRNA3//P2RX2//GRIN2B//PPP3CA//KCNIP2//TRDN//TMEM108//SLC26A1// |
| GO:0016579 | protein deubiquitination | Biological process | 11 | 291 | 368 | 17653 | 1.81330307784252 | 0.0422637488398687 | 0.868948251592414 | 1.3740319831886 | 0.0298913043478261 | COPS5//USP54//HIF1A//SMAD1//UCHL5//USP53//PSMD7//TNKS2//RNF135//OGT//ACTL6A// |
| GO:1902686 | mitochondrial outer membrane permeabilization involved in programmed cell death | Biological process | 4 | 63 | 368 | 17653 | 3.04572118702553 | 0.0423291449074726 | 0.868948251592414 | 1.3733605046353 | 0.0108695652173913 | PMAIP1//YWHAB//YWHAZ//SLC25A5// |
| GO:0035567 | non-canonical Wnt signaling pathway | Biological process | 7 | 154 | 368 | 17653 | 2.18045948616601 | 0.0427189366588577 | 0.868948251592414 | 1.36937956611382 | 0.0190217391304348 | FZD2//GNAO1//PPP3CA//PPP3CB//FZD3//PSMD7//SFRP1// |
| GO:0001977 | renal system process involved in regulation of blood volume | Biological process | 2 | 16 | 368 | 17653 | 5.99626358695652 | 0.0428949128087064 | 0.868948251592414 | 1.36759421061509 | 0.00543478260869565 | EMP2//CYP4F2// |
| GO:0034315 | regulation of Arp2/3 complex-mediated actin nucleation | Biological process | 2 | 16 | 368 | 17653 | 5.99626358695652 | 0.0428949128087064 | 0.868948251592414 | 1.36759421061509 | 0.00543478260869565 | BRK1//ARFIP2// |
| GO:0045072 | regulation of interferon-gamma biosynthetic process | Biological process | 2 | 16 | 368 | 17653 | 5.99626358695652 | 0.0428949128087064 | 0.868948251592414 | 1.36759421061509 | 0.00543478260869565 | FOXP3//EBI3// |
| GO:0045717 | negative regulation of fatty acid biosynthetic process | Biological process | 2 | 16 | 368 | 17653 | 5.99626358695652 | 0.0428949128087064 | 0.868948251592414 | 1.36759421061509 | 0.00543478260869565 | ACADL//INSIG2// |
| GO:0051571 | positive regulation of histone H3-K4 methylation | Biological process | 2 | 16 | 368 | 17653 | 5.99626358695652 | 0.0428949128087064 | 0.868948251592414 | 1.36759421061509 | 0.00543478260869565 | KMT2E//PAXIP1// |
| GO:0061298 | retina vasculature development in camera-type eye | Biological process | 2 | 16 | 368 | 17653 | 5.99626358695652 | 0.0428949128087064 | 0.868948251592414 | 1.36759421061509 | 0.00543478260869565 | NDP//HIF1A// |
| GO:0080182 | histone H3-K4 trimethylation | Biological process | 2 | 16 | 368 | 17653 | 5.99626358695652 | 0.0428949128087064 | 0.868948251592414 | 1.36759421061509 | 0.00543478260869565 | KMT2E//OGT// |
| GO:0006935 | chemotaxis | Biological process | 19 | 594 | 368 | 17653 | 1.53439741619089 | 0.0434943315473121 | 0.868948251592414 | 1.36156733932429 | 0.0516304347826087 | CXCL11//EFNA3//NCAM1//NRAS//CNTN2//VLDLR//UNC5A//TNFRSF8//TNFSF14//CH25H//FZD3//SEMA4G//AGTR1//PARVA//JAM3//DLX5//ZSWIM6//RALBP1//RHOH// |
| GO:0031334 | positive regulation of protein complex assembly | Biological process | 10 | 257 | 368 | 17653 | 1.86654119438335 | 0.0436817273900427 | 0.868948251592414 | 1.35970019582689 | 0.0271739130434783 | RHOH//CDKN1B//MAPRE1//TRABD2B//PMAIP1//ACTR3//ACTR2//ARFIP2//PAXIP1//BRK1// |
| GO:0060359 | response to ammonium ion | Biological process | 6 | 123 | 368 | 17653 | 2.34000530222694 | 0.0440254043082503 | 0.868948251592414 | 1.35629664698963 | 0.016304347826087 | CHRM1//SMPD1//GNAO1//CASP7//CHRNA3//LRRK2// |
| GO:0030900 | forebrain development | Biological process | 13 | 366 | 368 | 17653 | 1.70385631979092 | 0.0440276698026592 | 0.868948251592414 | 1.35627429929238 | 0.0353260869565217 | TMEM108//PAX5//UCHL5//LRRK2//FBXO45//DIXDC1//RTN4//CNTN2//OTP//DLX5//ALDH1A2//HIF1A//GNAO1// |
| GO:0045765 | regulation of angiogenesis | Biological process | 13 | 366 | 368 | 17653 | 1.70385631979092 | 0.0440276698026592 | 0.868948251592414 | 1.35627429929238 | 0.0353260869565217 | EFNA3//ABCC8//HIPK1//DDAH1//HIF1A//NOS3//RRAS//AGTR1//ITGA5//SMAD1//EMP2//SFRP1//PROK1// |
| GO:0042330 | taxis | Biological process | 19 | 595 | 368 | 17653 | 1.53181859700402 | 0.0441033169596219 | 0.868948251592414 | 1.35552874651655 | 0.0516304347826087 | CXCL11//RALBP1//RHOH//EFNA3//NCAM1//NRAS//CNTN2//VLDLR//UNC5A//TNFRSF8//TNFSF14//CH25H//FZD3//SEMA4G//AGTR1//PARVA//JAM3//DLX5//ZSWIM6// |
| GO:0002639 | positive regulation of immunoglobulin production | Biological process | 3 | 38 | 368 | 17653 | 3.78711384439359 | 0.0443008768365646 | 0.868948251592414 | 1.35358767780598 | 0.00815217391304348 | PAXIP1//TNFSF4//IL4R// |
| GO:0014904 | myotube cell development | Biological process | 3 | 38 | 368 | 17653 | 3.78711384439359 | 0.0443008768365646 | 0.868948251592414 | 1.35358767780598 | 0.00815217391304348 | P2RX2//C11ORF88//PPP3CA// |
| GO:0046676 | negative regulation of insulin secretion | Biological process | 3 | 38 | 368 | 17653 | 3.78711384439359 | 0.0443008768365646 | 0.868948251592414 | 1.35358767780598 | 0.00815217391304348 | PPP3CA//SFRP1//ABCC8// |
| GO:0090311 | regulation of protein deacetylation | Biological process | 3 | 38 | 368 | 17653 | 3.78711384439359 | 0.0443008768365646 | 0.868948251592414 | 1.35358767780598 | 0.00815217391304348 | FOXP3//LRRK2//BEX4// |
| GO:1901030 | positive regulation of mitochondrial outer membrane permeabilization involved in apoptotic signaling pathway | Biological process | 3 | 38 | 368 | 17653 | 3.78711384439359 | 0.0443008768365646 | 0.868948251592414 | 1.35358767780598 | 0.00815217391304348 | PMAIP1//YWHAB//YWHAZ// |
| GO:0031325 | positive regulation of cellular metabolic process | Biological process | 77 | 3074 | 368 | 17653 | 1.20159348391842 | 0.0446247581266805 | 0.868948251592414 | 1.35042412481886 | 0.209239130434783 | LRRK2//PRKAA1//PROK1//CD81//PAXIP1//VAMP3//PMAIP1//CHRNA3//HEYL//CASP7//TP53INP2//AGTR1//MICAL2//GDF9//BMP10//TTK//HIF1A//STARD4//ABHD5//CNTN2//CPEB3//LARP1//RBM3//DLX5//ELK1//FZD2//NDP//FOXP3//PPP3CB//KMT2E//SFRP1//PAX8//SKAP1//CREB5//CXCL11//PAXBP1//NOS3//NDFIP1//EMP2//OXSR1//NEK7//TNKS2//RNF217//INSIG2//IFNK//PRKAR2B//SMPD1//TNFRSF8//TNFAIP8L3//EBI3//DDAH1//CDKN1B//VLDLR//OGT//COPS5//PRDM4//EPAS1//RRP1B//ATP1B4//TLX2//MCIDAS//SMAD1//PAX5//PPP3CA//SIM1//VEZF1//CREB3L3//ITGA5//PRDM12//CALCOCO2//EFNA3//TRABD2B//FAM168A//TNFSF4//ITGA8//PIP4K2C//GRIN2B// |
| GO:0000045 | autophagosome assembly | Biological process | 5 | 93 | 368 | 17653 | 2.57903810191678 | 0.0450009988179414 | 0.868948251592414 | 1.34677784675118 | 0.0135869565217391 | LRRK2//PIP4K2C//UBXN2B//UBXN2A//TP53INP2// |
| GO:0045619 | regulation of lymphocyte differentiation | Biological process | 7 | 156 | 368 | 17653 | 2.15250487736901 | 0.0452579262425967 | 0.868948251592414 | 1.34430534967494 | 0.0190217391304348 | FOXP3//TNFSF4//CD46//HMGB3//SFRP1//IL4R//NDFIP1// |
| GO:0003206 | cardiac chamber morphogenesis | Biological process | 6 | 124 | 368 | 17653 | 2.32113429172511 | 0.0454896027010861 | 0.868948251592414 | 1.34208785621202 | 0.016304347826087 | PARVA//FZD2//HEYL//HIF1A//UBE4B//BMP10// |
| GO:0045834 | positive regulation of lipid metabolic process | Biological process | 6 | 124 | 368 | 17653 | 2.32113429172511 | 0.0454896027010861 | 0.868948251592414 | 1.34208785621202 | 0.016304347826087 | STARD4//AGTR1//ABHD5//CD81//TNFAIP8L3//PRKAA1// |
| GO:0099565 | chemical synaptic transmission, postsynaptic | Biological process | 6 | 124 | 368 | 17653 | 2.32113429172511 | 0.0454896027010861 | 0.868948251592414 | 1.34208785621202 | 0.016304347826087 | CHRNA3//LRRK2//P2RX2//GRIN2B//PPP3CA//TMEM108// |
| GO:0050768 | negative regulation of neurogenesis | Biological process | 10 | 259 | 368 | 17653 | 1.85212774886688 | 0.0456157623375205 | 0.868948251592414 | 1.34088506274446 | 0.0271739130434783 | LRRK2//TNFRSF8//BAG5//RTN4//CNTN2//DIXDC1//SEMA4G//TLX2//PPP3CA//ABCC8// |
| GO:0009966 | regulation of signal transduction | Biological process | 80 | 3215 | 368 | 17653 | 1.19365744810332 | 0.0465220331178208 | 0.868948251592414 | 1.33234131388004 | 0.217391304347826 | LRRK2//PRKAA1//PROK1//CD81//RIC8B//CD46//SKAP1//EBI3//FGF11//GDF9//BMP10//NDP//IFNK//CXCL11//TG//TNFSF4//TNFSF14//HIPK1//TTK//PIP4K2C//MGRN1//YWHAB//NREP//SMPD1//FOXP3//SFRP1//TRABD2B//CXXC4//ITGA8//HTRA4//SKIL//RASL11B//VWC2//HIF1A//ITGA5//RALGPS2//WDFY1//OGT//EPS8L2//ARHGEF6//CTH//NDFIP1//RHOH//RNF149//PMAIP1//TSPAN14//EVC//COPS5//LRRTM1//P2RX2//PAX5//FAM13A//RALBP1//ARHGAP36//RHOF//CGNL1//TMEM108//RRAS//TNFAIP8L3//RTN4//PALM//CNTN2//JPH1//TRDN//HEYL//CTNND2//PSMD7//DLX5//TNKS2//DIXDC1//LYPD6//YWHAZ//SLC25A5//PTPN18//NOS3//BAG5//LDOC1//EMP2//PDIA3//PPP1R2// |
| GO:0007423 | sensory organ development | Biological process | 17 | 521 | 368 | 17653 | 1.56524346991571 | 0.0465519843381922 | 0.868948251592414 | 1.33206180192733 | 0.046195652173913 | HIPK1//CTNS//ACTL6A//ALDH1A2//DLX5//INSIG2//PAX8//FZD3//ITGA8//KCNQ4//GPD2//CDKN1B//FZD2//HIF1A//NDP//SKIL//BFSP1// |
| GO:0035794 | positive regulation of mitochondrial membrane permeability | Biological process | 4 | 65 | 368 | 17653 | 2.95200668896321 | 0.0466054009778556 | 0.868948251592414 | 1.33156375114346 | 0.0108695652173913 | PMAIP1//YWHAB//YWHAZ//SLC25A5// |
| GO:0051926 | negative regulation of calcium ion transport | Biological process | 4 | 65 | 368 | 17653 | 2.95200668896321 | 0.0466054009778556 | 0.868948251592414 | 1.33156375114346 | 0.0108695652173913 | TRDN//SESTD1//GNAO1//NOS3// |
| GO:0097305 | response to alcohol | Biological process | 8 | 191 | 368 | 17653 | 2.00921921238334 | 0.0472404100756892 | 0.868948251592414 | 1.3256863411001 | 0.0217391304347826 | ACTR2//ALAD//GRIN2B//CLDN18//PRKAA1//SFRP1//TNFSF4//LARP1// |
| GO:0042088 | T-helper 1 type immune response | Biological process | 3 | 39 | 368 | 17653 | 3.69000836120401 | 0.0472761649026315 | 0.868948251592414 | 1.32535776117035 | 0.00815217391304348 | IL4R//TNFSF4//EBI3// |
| GO:0046638 | positive regulation of alpha-beta T cell differentiation | Biological process | 3 | 39 | 368 | 17653 | 3.69000836120401 | 0.0472761649026315 | 0.868948251592414 | 1.32535776117035 | 0.00815217391304348 | FOXP3//TNFSF4//IL4R// |
| GO:0010033 | response to organic substance | Biological process | 80 | 3218 | 368 | 17653 | 1.19254465371416 | 0.0473797621127416 | 0.868948251592414 | 1.32440712414054 | 0.217391304347826 | PPP3CA//HSPA13//FERMT2//GDF9//SMAD1//SKIL//CHRM1//ATP6V1G2//POLR2E//PTBP1//NOS3//P2RX2//CDKN1B//ALAD//HIPK1//SFRP1//NREP//HIF1A//LRRTM1//IL4R//IFNK//YWHAZ//CPEB3//UBE4B//DLX5//BMP10//UBE2D3//ITGA8//HTRA4//RASL11B//VWC2//ITGA5//CTH//CREB3L3//PRKAA1//ALDH1A2//CXCL11//ABCC8//TNFRSF8//INSIG2//OGT//PSMD7//TNFSF4//TNFSF14//GNAO1//PGGT1B//PPP3CB//CALCOCO2//ACTR2//KDELR3//TLN1//GOSR2//DNAJB12//AGTR1//SMPD1//PMAIP1//GRIN2B//CLDN18//TMEM108//NCAM1//HEYL//EBI3//SKP1//MRC1//MAPK4//PTPN18//ACTR3//VAMP3//PAX8//PRKAR2B//ELK1//CD81//CASP7//LARP1//CHRNA3//LRRK2//COPS5//UBXN2A//EIF4A2//SLC25A5// |
| GO:0046883 | regulation of hormone secretion | Biological process | 10 | 261 | 368 | 17653 | 1.83793519906713 | 0.0476069855811467 | 0.868948251592414 | 1.32232931667546 | 0.0271739130434783 | AGTR1//SLC25A5//PPP3CB//ABCC8//CHRNA3//HIF1A//PPP3CA//SFRP1//PAX8//GDF9// |
| GO:0030154 | cell differentiation | Biological process | 100 | 4124 | 368 | 17653 | 1.1631937123097 | 0.047784013043322 | 0.868948251592414 | 1.32071737971415 | 0.271739130434783 | GDF9//PAXIP1//DLX5//TP53INP2//SFRP1//SMAD1//HIF1A//SEMA4G//FBXO45//MARK1//FZD3//VEZF1//OTP//STON2//MLF1//JAM3//FOXP3//RRAS//NDFIP1//HEYL//KRT2//PAX8//STRBP//LRRC38//RTN4//PARD6B//EFNA3//NCAM1//NRAS//CNTN2//VLDLR//UNC5A//TNFRSF8//AGTR1//ZNF804A//LRRK2//BAG5//ALDH1A2//COPS2//HIPK1//FZD2//IFNK//RHOH//PPP3CB//EPAS1//KMT2E//NREP//UBE4B//GNAO1//PRDM12//TMEM108//KRTAP1-1//FERMT2//PARVA//FERMT3//ITGA8//VAMP3//PAX5//ITGA5//MAEA//TNFSF4//CD46//CD8A//KCNIP2//BMP10//HMGB3//SKIL//IL4R//TNFSF14//DIXDC1//VWC2//BFSP1//MCMDC2//P2RX2//C11ORF88//PPP3CA//CHRNA3//TLX2//PTBP1//ABCC8//NPTX1//CTNND2//CPEB3//ACTR2//PSMD7//ZSWIM6//CDKN1B//MCIDAS//CTH//CLDN18//EMP2//ELK1//FOXG1//KIF2A//MEA1//ABHD5//ASF1B//PURA//SIM1//SEPT7// |
| GO:0003205 | cardiac chamber development | Biological process | 7 | 158 | 368 | 17653 | 2.12525798018712 | 0.0478919459581982 | 0.868948251592414 | 1.31973751623077 | 0.0190217391304348 | PARVA//FZD2//HEYL//HIF1A//UBE4B//PAX8//BMP10// |
| GO:0050864 | regulation of B cell activation | Biological process | 7 | 158 | 368 | 17653 | 2.12525798018712 | 0.0478919459581982 | 0.868948251592414 | 1.31973751623077 | 0.0190217391304348 | CD81//HMGB3//SFRP1//PAXIP1//FOXP3//NDFIP1//TNFSF4// |
| GO:0055067 | monovalent inorganic cation homeostasis | Biological process | 7 | 158 | 368 | 17653 | 2.12525798018712 | 0.0478919459581982 | 0.868948251592414 | 1.31973751623077 | 0.0190217391304348 | AGTR1//ATP1B4//LRRK2//SLC26A1//SCN7A//CYP4F2//ATP6V1G2// |
| GO:0002710 | negative regulation of T cell mediated immunity | Biological process | 2 | 17 | 368 | 17653 | 5.64354219948849 | 0.0479586099843858 | 0.868948251592414 | 1.31913341283047 | 0.00543478260869565 | PPP3CB//FOXP3// |
| GO:0009950 | dorsal/ventral axis specification | Biological process | 2 | 17 | 368 | 17653 | 5.64354219948849 | 0.0479586099843858 | 0.868948251592414 | 1.31913341283047 | 0.00543478260869565 | CXXC4//SFRP1// |
| GO:0030502 | negative regulation of bone mineralization | Biological process | 2 | 17 | 368 | 17653 | 5.64354219948849 | 0.0479586099843858 | 0.868948251592414 | 1.31913341283047 | 0.00543478260869565 | HIF1A//STATH// |
| GO:0042095 | interferon-gamma biosynthetic process | Biological process | 2 | 17 | 368 | 17653 | 5.64354219948849 | 0.0479586099843858 | 0.868948251592414 | 1.31913341283047 | 0.00543478260869565 | FOXP3//EBI3// |
| GO:0042249 | establishment of planar polarity of embryonic epithelium | Biological process | 2 | 17 | 368 | 17653 | 5.64354219948849 | 0.0479586099843858 | 0.868948251592414 | 1.31913341283047 | 0.00543478260869565 | FZD2//SFRP1// |
| GO:0045063 | T-helper 1 cell differentiation | Biological process | 2 | 17 | 368 | 17653 | 5.64354219948849 | 0.0479586099843858 | 0.868948251592414 | 1.31913341283047 | 0.00543478260869565 | IL4R//TNFSF4// |
| GO:0046641 | positive regulation of alpha-beta T cell proliferation | Biological process | 2 | 17 | 368 | 17653 | 5.64354219948849 | 0.0479586099843858 | 0.868948251592414 | 1.31913341283047 | 0.00543478260869565 | EBI3//TNFSF4// |
| GO:0071636 | positive regulation of transforming growth factor beta production | Biological process | 2 | 17 | 368 | 17653 | 5.64354219948849 | 0.0479586099843858 | 0.868948251592414 | 1.31913341283047 | 0.00543478260869565 | FOXP3//CD46// |
| GO:1904355 | positive regulation of telomere capping | Biological process | 2 | 17 | 368 | 17653 | 5.64354219948849 | 0.0479586099843858 | 0.868948251592414 | 1.31913341283047 | 0.00543478260869565 | NEK7//TNKS2// |
| GO:0001944 | vasculature development | Biological process | 23 | 761 | 368 | 17653 | 1.44981931668857 | 0.0483802107813999 | 0.868948251592414 | 1.31533224384931 | 0.0625 | EPAS1//HIF1A//ITGA5//NOS3//RTN4//VEZF1//JAM3//PROK1//ALDH1A2//PAXIP1//PPP3CB//PARVA//EFNA3//ABCC8//EMP2//SFRP1//HIPK1//DDAH1//RRAS//NDP//AGTR1//SMAD1//CTH// |
| GO:0033365 | protein localization to organelle | Biological process | 25 | 842 | 368 | 17653 | 1.42429063823195 | 0.0484508644068171 | 0.868948251592414 | 1.31469847034156 | 0.0679347826086956 | PDIA3//SPRN//PPP3CA//NUP214//PMAIP1//KDELR3//GOSR2//TIMM23//TOMM34//PEX5//TOMM40L//LRRK2//TTK//MAPRE1//SPCS2//SKP1//CD81//TULP4//TNKS2//CLDN18//YWHAB//YWHAZ//PRKAA1//UBE2D3//RTN4// |
| GO:1903747 | regulation of establishment of protein localization to mitochondrion | Biological process | 6 | 126 | 368 | 17653 | 2.28429089026915 | 0.0485081381415822 | 0.868948251592414 | 1.31418539431549 | 0.016304347826087 | PMAIP1//YWHAB//YWHAZ//LRRK2//PRKAA1//UBE2D3// |
| GO:0002312 | B cell activation involved in immune response | Biological process | 4 | 66 | 368 | 17653 | 2.90727931488801 | 0.048830335249323 | 0.868948251592414 | 1.31131029401546 | 0.0108695652173913 | PAXIP1//FOXP3//NDFIP1//TNFSF4// |
| GO:0045766 | positive regulation of angiogenesis | Biological process | 8 | 193 | 368 | 17653 | 1.98839828790268 | 0.0496660672292984 | 0.868948251592414 | 1.30394022792978 | 0.0217391304347826 | AGTR1//ITGA5//SMAD1//HIPK1//DDAH1//HIF1A//NOS3//RRAS// |
